# Kinetic‐Controlled Crystallization of *α*‐FAPbI_3_ Inducing Preferred Crystallographic Orientation Enhances Photovoltaic Performance

**DOI:** 10.1002/advs.202300798

**Published:** 2023-03-30

**Authors:** Sooeun Shin, Seongrok Seo, Seonghwa Jeong, Anir S. Sharbirin, Jeongyong Kim, Hyungju Ahn, Nam‐Gyu Park, Hyunjung Shin

**Affiliations:** ^1^ Department of Energy Science Sungkyunkwan University Suwon 440‐746 Republic of Korea; ^2^ SKKU Institute of Energy Science and Technology (SIEST) Sungkyunkwan University Suwon 440‐746 Republic of Korea; ^3^ Department of Physics University of Oxford Clarendon Laboratory Oxford OX1 3PU UK; ^4^ Pohang Accelerator Laboratory Pohang Kyungbuk 37673 Republic of Korea; ^5^ School of Chemical Engineering Sungkyunkwan University Suwon 440‐746 Republic of Korea

**Keywords:** crystallization kinetic, formamidinium lead triiodide, methylammonium chloride additive, perovskite solar cell, photovoltaic performance, preferred orientation

## Abstract

Crystallization kinetic controls the crystallographic orientation, inducing anisotropic properties of the materials. As a result, preferential orientation with advanced optoelectronic properties can enhance the photovoltaic devices' performance. Although incorporation of additives is one of the most studied methods to stabilize the photoactive *α*‐phase of formamidinium lead tri‐iodide (*α*‐FAPbI_3_), no studies focus on how the additives affect the crystallization kinetics. Along with the role of methylammonium chloride (MACl) as a “stabilizer” in the formation of *α*‐FAPbI_3_, herein, the additional role as a “controller” in the crystallization kinetics is pointed out. With microscopic observations, for example, electron backscatter diffraction and selected area electron diffraction, it is examined that higher concentration of MACl induces slower crystallization kinetics, resulting in larger grain size and [100] preferred orientation. Optoelectronic properties of [100] preferentially oriented grains with less non‐radiative recombination, a longer lifetime of charge carriers, and lower photocurrent deviations in between each grain induce higher short‐circuit current density (*J*_sc_) and fill factor. Resulting MACl40 mol% attains the highest power conversion efficiency (PCE) of 24.1%. The results provide observations of a direct correlation between the crystallographic orientation and device performance as it highlights the importance of crystallization kinetics resulting in desirable microstructures for device engineering.

## Graphical abstract

Incorporating methylammonium chloride (MACl) as an additive not only “stabilizes” the formation of formamidinium lead tri‐iodide (*α*‐FAPbI_3_), but also “controls” the crystallization kinetics. As higher concentration of MACl induces slower crystallization, it results in larger grain size and [100] preferred orientation. With improved optoelectronic properties of [100] preferentially oriented grains, the highest power conversion efficiency of 24.1% is attained.

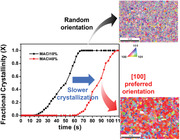

## 1 Introduction

Physical properties of the polycrystalline materials are mostly determined by their microstructure. As the crystallization process can determine the microstructure, the nucleation, and growth can also control whether the materials will be resulted in single crystalline or polycrystalline.^[^ ^1^ ^]^ Along with the morphological changes, anisotropic properties of the materials can also be controlled.^[^ ^2^, ^3^, ^4^, ^5^ ^]^ One of the most well‐known studies on the crystallization kinetics is so‐called, t‐t‐t, time‐temperature transformation in the binary alloy of Fe‐C system.^[^ ^6^ ^]^ With the concentration ratio between Fe and C the kinetics can control the microstructure of various alloys and their composition, allowing to achieve the profitable mechanical properties to be used in many applications. The control of microstructures induced by crystallization kinetics is also found in hybrid organic–inorganic metal halides. For example, formation of the intermediates via Lewis base additive prolongs the phase transformation and therefore slows down the crystallization kinetics.^[^ ^7^, ^8^ ^]^ With the enhanced crystal quality, studies reported improvement in optoelectronic properties.^[^ ^5^, ^7^, ^9^, ^10^ ^]^ Incorporation of cations and/or anions was also reported to control the crystallization kinetics.^[^ ^11^, ^12^ ^]^ As a result, regulation of crystallization kinetics can manipulate the physical properties of the materials and therefore modulate the device performance.

In recent studies, perovskite solar cells (PSCs) have boosted the certified record power conversion efficiency (PCE) up to 25.8%^[^ ^13^ ^]^ by processing engineering,^[^ ^14^, ^15^, ^16^ ^]^ compositional engineering, that is, triple cations of methylammonium (MA), formamidinium (FA), and Cs,^[^ ^17^ ^]^ and interfacial engineering.^[^ ^18^, ^19^ ^]^ The latest champion PCEs are all based on formamidinium lead triiodide (FAPbI_3_) with different additives, for example, methylammonium chloride (MACl),^[^ ^13^, ^20^, ^21^ ^]^ MAPbBr_3_,^[^ ^20^ ^]^ formamidinium formate (FAHCOO),^[^ ^21^ ^]^ and methylenediamine dihydrochloride (MDACl_2_),^[^ ^13^ ^]^ reaching 25.2%, 25.6%, and 25.8%. Unfortunately, FAPbI_3_ often shows a phase transition between 1) a non‐perovskite yellow phase (*δ*‐FAPbI_3_, space group of P6_3_mc) with an indirect bandgap of ≈2.48 eV, which is synthesized at 220 K, and 2) a perovskite black phase (*α*‐FAPbI_3_, Pm$\bar{3}$m) with a direct bandgap of ≈1.45 eV, which is synthesized at 390 K.^[^ ^22^ ^]^ Although *α*‐FAPbI_3_ is the most thermodynamically stable photoactive cubic phase, it can chemically stabilize as *δ*‐FAPbI_3_ in the ambient environment.^[^ ^23^ ^]^ Therefore, the phase stabilization of photoactive *α*‐FAPbI_3_ is of paramount importance for the fabrication of highly efficient FAPbI_3_‐based PSCs. Studies have reported partial substitution of FA^+^ species with MA^+^ or Cs^+^ to suppress the formation of *δ*‐FAPbI_3_ and stabilize *α*‐FAPbI_3_ in the ambient environment.^[^ ^17^, ^24^, ^25^, ^26^ ^]^ The incorporation of additives, such as methylammonium iodide (MAI)^[^ ^27^ ^]^ and cesium iodide (CsI),^[^ ^17^, ^28^, ^29^, ^30^ ^]^ is indeed substituting the A‐site cations, FA^+^, leading to a bandgap energy increase and thereby narrows the absorption spectrum.^[^ ^31^ ^]^ On the other hand, incorporating additive such as MACl,^[^ ^31^, ^32^, ^33^, ^34^ ^]^ less affects the optical bandgap energy regardless of the amount of additives,^[^ ^33^, ^35^ ^]^ which implies incorporating MACl induces different processes in the stabilization and/or formation of *α*‐FAPbI_3_ other than MA ion substitution. As a result, the state‐of‐the‐art PSCs are based on stabilized and well‐ordered *α*‐FAPbI_3_ absorber films with MACl. Wang et al. demonstrated that additions of FACl and MACl into the FAPbI_3_ precursor solution assisted the crystallization of phase‐pure *α*‐FAPbI_3_ via the formation of intermediate mixtures.^[^ ^36^ ^]^ Qing et al. reported synergistic effects of dimethylsulfoxide (DMSO) and MACl additives assisting crystallization and leading to single‐crystal‐like films.^[^ ^37^ ^]^ Kim et al. also investigated the role of MACl in the formation of the *α*‐FAPbI_3_ perovskite structure.^[^ ^35^ ^]^ Interestingly, numerous studies reported grain growth and preferred orientation as a result of adding not only MACl^[^ ^38^, ^39^, ^40^, ^41^ ^]^ but also any chloride‐based additive (e.g., FACl,^[^ ^42^, ^43^, ^44^ ^]^ CsCl,^[^ ^42^, ^43^, ^45^, ^46^ ^]^ RbCl,^[^ ^42^ ^]^ and ethylamine chloride (EACl^[^ ^47^ ^]^)). Most of studies with MACl as an additive just addressed stabilization process of the *α*‐FAPbI_3._ While only a few studies have focused on how MACl results the preferential growth in a specific direction.^[^ ^41^, ^48^, ^49^, ^50^ ^]^ No studies were found in the literature on how MACl additives affect the development of nanoscale materials heterogeneity during crystallization despite stabilizing the cubic phase of *α*‐FAPbI_3_. Additionally, observations of the effect of large grains with a preferred orientation on device performance were limited to the macroscopic view only.^[^ ^38^, ^39^, ^40^, ^51^ ^]^

As many studies focused on the role of MACl as a “stabilizer” in the formation of *α*‐FAPbI_3_, herein, we pointed out the additional role as a “controller” in the crystallization kinetics. We examined how the additives affect the overall crystallization process and demonstrate higher concentration of the additive may slow down the crystallization, resulting in enlarged grain size and preferred orientation which leads to PCE improvements. Therefore, despite most studies have ascribed the higher PCE to higher concentration of the additive incorporating within FAPbI_3_, we achieved PCE improvement through slowing down the crystallization with lower heat treatment temperatures regardless of the low concentration of additives. To understand the role of MACl‐incorporated crystallization process of halide perovskites, we should focus on the crystallization kinetics, that is, nucleation and growth. As the grain size and crystal orientation can be controlled by regulating nucleation and crystal growth; fast nucleation, a short period of time resulting in a small number of nuclei, and sluggish crystal growth are the key to forming a large grain size and an ordered crystal orientation.^[^ ^52^, ^53^ ^]^ Fast nucleation requires supersaturation of the precursor solute; therefore, anti‐solvent,^[^ ^54^, ^55^, ^56^ ^]^ gas‐assisted,^[^ ^57^ ^]^ and thermal annealing^[^ ^58^ ^]^ methods were used to control the rate of solvent evaporation. Slow crystallization requires suppressed growth rates; therefore, Lewis acid‐base adducts,^[^ ^59^, ^60^ ^]^ additives,^[^ ^61^, ^62^, ^63^, ^64^, ^65^ ^]^ and solvent annealing^[^ ^66^, ^67^ ^]^ were used to generate a preferential orientation of the lowest‐surface‐energy facet among the crystal planes.^[^ ^65^ ^]^ Based on the above perspectives, by using diethyl ether as an anti‐solvent, the fast nucleation can be obtained and the use of MACl as an additive slows down the crystallization kinetics of *α*‐FAPbI_3_, hence enlarging the grain size and ordering the crystal orientation to the crystal plane of the lowest surface energy. Controlling the crystallization kinetics can be proposed to enhance the photovoltaic (PV) performance, thus leading to desirable microstructures of *α*‐FAPbI_3_ absorber films.^[^ ^68^ ^]^

Here, we demonstrate that the crystallization kinetics can control the crystallographic orientation as well as the grain size in *α*‐FAPbI_3_ films and thus impact the PV performance by adjusting the concentration of MACl additive to 10, 20, 30, and 40 mol%, and a heat treatment. As we recorded the crystallization process, Avrami exponent increases with the higher concentration of MACl indicating slower crystallization kinetics. With higher concentration of MACl, *α*‐FAPbI_3_ thin films with [100] preferentially oriented grains were examined by electron backscatter diffraction (EBSD), selected area electron diffraction (SAED), and synchrotron‐based grazing‐incidence X‐ray diffraction (GIXRD). With confocal photoluminescence (PL) and time‐resolved PL (TRPL) microscopic mapping, the *α*‐FAPbI_3_ thin films with 40% MACl showed higher PL intensity and longer PL lifetime than those with 10%, 20%, and 30% MACl. Conductive atomic force microscopy (C‐AFM) indicated much more homogeneous photocurrent generation along the surface of (100) preferentially oriented layers. Less photocurrent deviation between grains was found in the *α*‐MACl40% FAPbI_3_ films. We clearly demonstrated, in this study, the role of nanoscale materials heterogeneities, which can be controlled by crystallization kinetics, on macroscopic device performance. The device performance is enhanced by improving the short‐circuit current density (*J_sc_*) and fill factor (FF). The improved optoelectronic properties of MACl40% films, resulted in the highest PCE of 24.1%. Which was derived from the slower crystallization kinetics resulting larger grain size and [100] preferentially oriented grain growth. The MACl10% films showed the smallest grain size of ≈340 nm on average with random orientations, resulting in the lowest PCE of 18.4%. We further propose that crystallization kinetics can be controlled by simple heat treatment modification based on the same amount of additives, which induced enlargement of the grain size along with the [100] preferred orientation. As a result, the PCE of MACl10% was improved from 18.4% to 21.8% (champion PCE of 22.5%). This not only confirms the observations of a direct correlation between the crystallographic orientation and device performance but also highlights the importance of crystallization kinetics resulting in desirable microstructures for device engineering.

## 2 Results

### 2.1 Crystallographic Orientation of *α*‐FAPbI_3_

**Figure 1:**
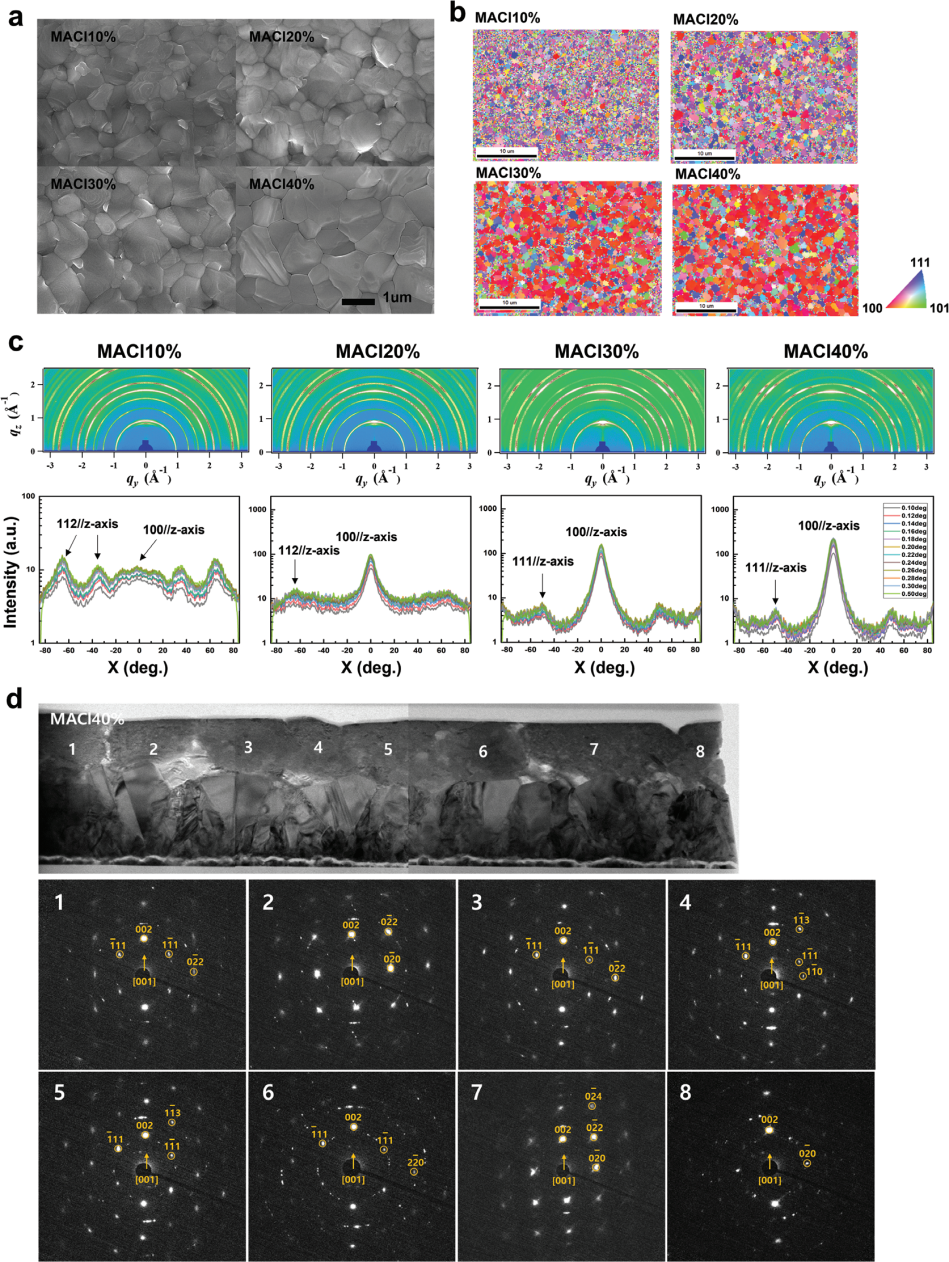
Structural characterizations of *α*‐FAPbI_3_ thin films. a) SEM images of *α*‐FAPbI_3_ thin films with different concentrations of MACl: 10%, 20%, 30%, and 40%. As the amount of MACl increases, the average grain size also increases. b) Crystallographic orientational information of each *α*‐FAPbI_3_ thin film obtained by EBSD. The red regions indicate the (100) orientation, and the blue and purple regions indicate the (111) and (112) orientations perpendicular to the substrate. As the EBSD image of MACl40% shows more red regions than the EBSD image of MACl10%, MACl40% thin films exhibited a preferred (100) orientation, whereas MACl10% thin films were randomly oriented. c) Synchrotron‐based GIXRD patterns and corresponding intensity distributions of *α*‐FAPbI_3_ thin films with 10, 20, 30, and 40% MACl. The peak positions for all of the *α*‐FAPbI_3_ thin films were well indexed to the cubic structure. As the amount of MACl increases, the 100//*z*–axis peak appears and is strongly enhanced, which demonstrates that the crystal structure of MACl40% is preferentially oriented in the [100] direction. d) Cross‐sectional HRTEM image of the MACl40% thin film and its corresponding SAED patterns taken from each grain (numbered ≈1–8). The HRTEM specimen was prepared using a focused ion beam (FIB). Although the SAED patterns of MACl40% also indicated cubic *α*‐FAPbI_3_ in the Pm$\bar{3}$m space group, the arrow marked in the SAED patterns of each grain has the same [100] axis “face‐up” relative to the substrate.

Stabilized *α*‐FAPbI_3_ thin films were synthesized by incorporating different concentrations of MACl: 10, 20, 30, and 40 mol%. The surface morphologies of the *α*‐FAPbI_3_ thin films with well‐developed and dense crystalline grains were observed by scanning electron microscopy (SEM), as shown in **Figure** **1**a. The average grain size of ≈340 nm for MACl10% thin films increased to 460 (MACl20%), 800 (MACl30%), and 1,040 nm (MACl40%), as shown in the histograms of the grain size distributions for ≈100 grains each (Figure S1, Supporting Information). An increase in the average grain size was often found in early studies that used chloride precursors.^[^ ^40^, ^47^, ^49^, ^51^, ^69^, ^70^, ^71^ ^]^ Analyzing the local crystallographic orientation with EBSD in Figure 1b, MACl40% not only has the largest average grain size of ≈1 µm (Figure 1a) but also has a strong preferred orientation along the [100] direction normal to the surface of films. Corresponding inverse pole figures are shown in Figure S2, Supporting Information. The EBSD images were generated from the projection of the sample coordinate system (*xyz*) into the crystal coordinate system (*abc*).^[^ ^72^, ^73^, ^74^ ^]^ Comparing the lowest concentration, MACl10%, to the highest concentration, MACl40%, the amount of MACl affects the grain orientation, as MACl40% appears to have highly (100)‐oriented surfaces, whereas MACl10% appears to have random but partly (112)‐oriented surfaces. This observation corresponds to the synchrotron‐based GIXRD patterns in Figure 1c. The peak positions for all MACl samples indicate the cubic structure of Pm$\bar{3}$m space group. Strong peaks of the family of planes were observed in MACl30 and 40% thin films, which are also shown in the intensity distributions in Figure 1c (bottom row). Throughout the samples, the 112//*z*–axis peak appears in MACl10% and starts to disappear, while the 100//*z*–axis peak becomes the major peak in MACl40%. This surface orientation tendency throughout the samples is also demonstrated by the (100) orientation degree shown in Figure S3, Supporting Information, which was statistically obtained. As all the XRD patterns of the MACl samples indicate the formation of *α*‐FAPbI_3_ phase, the (100) orientation degree was calculated as the XRD peak intensity ratio of (100) and (111) from *α*‐FAPbI_3_ (I_(100)_/I_(111)_, Figure S3b, Supporting Information). Examining the EBSD image in Figure 1b, although the majority of the grains are oriented in the [100] direction, some grains show different crystal orientations, identified by different colors. Microscopic information of the individual grains was also obtained by cross‐sectional high‐resolution transmission electron microscopy (HRTEM) of MACl10%, as shown in Figure S4, Supporting Information, and MACl40%, as shown in Figure 1d. From the SAED patterns taken from each grain, the direction relative to the substrate was identified. Corresponding to the previous examinations in Figure 1 and Figures S2,S3, Supporting Information, MACl10% thin films showed that the “face‐up” direction from the substrate varies in each grain (Figure S4, Supporting Information). A range of axes, [111], [112], [113], and [100], was observed, as MACl10% was demonstrated to have a random orientation. On the other hand, MACl40% thin films only showed the [100] direction “face‐up” relative to the substrate in all of the grains (Figure 1d). Compared to MACl10% in Figure S3, Supporting Information, MACl40% demonstrates preferred growth alignment along the [100] axis, which is said to be “(100)‐oriented” growth. The overall surface orientation is from a collection of each grain orientation. We must consider this perspective throughout the study, as we further investigate the direct correlation between the crystallographic orientation and optoelectronic properties.

### 2.2 Role of MACl in Crystallizing *α*‐FAPbI_3_

Before we discuss the device performance, we note that since we synthesized the *α*‐FAPbI_3_ thin films with different MACl concentrations, we must consider how altering the MACl concentration results in the overall produced *α*‐FAPbI_3_. Kim et al. investigated the effects of MA cations incorporated into the perovskite structure using density functional theory (DFT).^[^ ^35^ ^]^ Incorporation of MA cations causes shrinkage of the cubo‐octahedral volume, resulting in stabilized *α*‐FAPbI_3_. Here, we investigated the incorporation of MA cations in MACl10, 20, 30, and 40 mol% thin films by using liquid‐state nuclear magnetic resonance (NMR), as shown in Figures S5 and S6, Supporting Information. Peaks corresponding to the C‐*H* proton from formamidine iodide (FAI) were observed in all of the MACl thin films in Figure S6, Supporting Information. Using the ^1^H‐NMR spectra of MAI and MACl as references (Figure S5, Supporting Information), no apparent peaks corresponding to the N‐*H* or C‐*H* proton in MACl were found throughout the samples. This indicated that irrespective of the MACl concentration, the remaining MACl within *α*‐FAPbI_3_ was undetectable. Furthermore, the amount of MA cations incorporated throughout the MACl samples may not vary to a large extent compared to the weight percentage difference of 10, 20, 30, and 40 mol% MACl in the precursor. This is consistent with Zhu et al., who quantified the relative amounts of FA and MA incorporated in a perovskite by liquid‐state ^1^H‐NMR.^[^ ^33^ ^]^ They reported that only a minimum amount of MA cations, with an x value in FA_1‐_ *_x_* MA*_x_* PbI_3_ equal to 0.06 ± 0.01, is necessary to stabilize *α*‐FAPbI_3_. X‐ray photoelectron spectroscopy (XPS) was also performed to examine the chemical components of *α*‐FAPbI_3_ in MACl10 and 40% thin films (Figure S7, Supporting Information). In both films, peaks corresponding to FA were obtained in the C 1s and N 1s peak regions.^[^ ^35^, ^75^ ^]^ No apparent Cl 2p peak was observed. Despite the presence of Cl ions in the precursor, the remaining Cl ions were also undetectable in the resulting *α*‐FAPbI_3_. As reported, Cl ions evaporate away during the annealing process in synthesizing *α*‐FAPbI_3_.^[^ ^34^, ^35^, ^36^, ^76^, ^77^ ^]^ Dar et al. reported that no evidence of Cl ions was found using scanning transmission electron microscopy‐energy dispersive spectroscopy (STEM‐EDS).^[^ ^76^ ^]^ In addition to stabilizing *α*‐FAPbI_3_, how the MACl additive affects the crystallization kinetics was investigated. The fractional crystallization curve for the isothermal crystallization process over time is shown in Figure S8, Supporting Information. The crystallization processes of both MACl10% and MACl40% were recorded. Nucleation and growth were detected after 12 and 55 s for MACl10% and MACl40%, respectively. It indicates that a higher concentration of MACl induces slower crystallization kinetics. With the Avrami plot of fractional crystallization, the Avrami exponent was obtained from the linear slope, *n*. As the Avrami exponent of MACl40% (*n* = 3.7) is higher than that of MACl10% (*n* = 2.6), the crystallization dimension increases with the decrease in the crystallization rate. This may be explained by the fact that a higher concentration of MACl induces a slower rate of solvent evaporation. With the slower crystallization kinetic, the growth of the (100) plane which has the lowest surface energy in the cubic structure, becomes dominant.^[^ ^78^ ^]^

To examine the structural evolution of the crystallization process, Ex situ XRD was conducted (Figure S9, Supporting Information). Although both MACl10% and MACl40% samples undergo an intermediate phase throughout the process, MACl40% shows slower reaction toward the intermediate phase to the crystalline FAPbI3. We note that to reveal the possible chemical reactions involved in the perovskite formation process, it requires identification for the unknown peaks of the intermediate phase which will be addressed in future studies. However, along with the intermediate phase, PbI_2_ was also related to the process. As the MACl40% XRD patterns show (001) peak of 2H PbI_2_ phase which decreases throughout the process. This result is consistent with previous research where Fei et al. examined chloride precursor to slow down the delivery speed of PbI_2_ from the intermediate phase CH_3_NH_3_PbI_2_Cl.^[^ ^40^ ^]^ As a result, higher concentration of the chloride within the MACl40% retards the transformation to crystalline FAPbI_3_. This results in the larger grain size and the ordered crystal orientation in Figure 1. Based on the above observations, the role of the MACl additive in *α*‐FAPbI_3_ is twofold: 1) stabilizing cubic *α*‐FAPbI_3_ phase by incorporating a minimal amount of MA cations and 2) controlling the surface morphology and orientation affected by the crystallization kinetics. Therefore, as we change the concentration of MACl, apparent variable conditions will be the surface morphology and orientation.

### 2.3 Device Performance of (100)‐Oriented *α*‐FAPbI_3_

*α*‐FAPbI_3_ powder was pre‐synthesized using a 1 m solution of PbI_2_ and FAI at an equal molar ratio in 10 mL of 2‐methoxyethanol, which was filtered and heated to 150 °C. The precursor solution (1.6 m FAPbI_3_) was prepared in a mixture of dimethylformamide (DMF) and DMSO (4:1 v/v), while MACl was added to the solution at concentrations of 10, 20, 30, and 40 mol%. Atomic layer deposition (ALD) was used to deposit a SnO_2_ compact layer, which was followed by a SnO_2_ nanoparticle coating as the electron transport layer (ETL).^[^ ^79^, ^80^, ^81^, ^82^ ^]^ 15 mm solution of *n*‐octylammonium iodide (OAI) and 16 mm solution of 4‐methoxy‐phenethylammonium iodide (4MEO‐PEAI) in 2‐propanol (IPA) were used as the passivation layer, which was followed by Spiro‐OMeTAD as the hole transport layer (HTL). PSCs with the structure FTO/ALD‐SnO_2_/SnO_2_ NP/*α*‐FAPbI_3_/OAI/Spiro‐OMeTAD/Au were fabricated.

**Figure 2:**
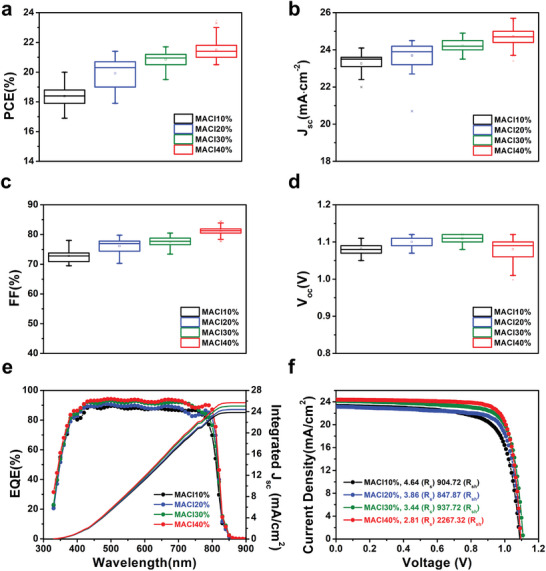
PV performance of *α*‐FAPbI_3_ thin films. a–d) Box charts of statistical PV parameters for *α*‐FAPbI_3_‐based PSCs with different concentrations of MACl: a) PCE in %, b) short‐circuit current density, *J*_sc_, c) fill factor, FF, and d) open‐circuit voltage, *V*_oc_. e) EQE spectra of *α*‐FAPbI_3_‐based PSCs. The integrated *J*_sc_ was extracted from the EQE spectrum. f) *J*–*V* curves of *α*‐FAPbI_3_‐based PSCs. The series resistance (*R*_s_) and shunt resistance (*R*_sh_) were extracted from a reverse scan of the *J*–*V* curves by measuring the negative inverse slope near *V*_oc_ and *J*_sc_, respectively. For all the device performance tests, PSCs with the structure FTO/ALD‐SnO_2_/SnO_2_ NP/*α*‐FAPbI_3_/OAI/Spiro‐OMeTAD/Au were fabricated. For each sample, 35 cells were used to collect the statistical PV parameters.

To investigate the impact of different surface orientations on the device performance, we compared the PV parameters of the PSCs. The PCE, short‐circuit current density (*J*_sc_), FF, and open‐circuit voltage (*V*_oc_) of *α*‐FAPbI_3_‐based PSCs with various concentrations of MACl are shown in **Figure** **2**a–d. The corresponding distribution parameters are summarized in **Table** **1**. The statistical distribution of the parameters provides a clear view of how the MACl concentration affects the overall device performance. Although *V*_oc_ does not follow the tendency of the MACl concentration, as the MACl concentration increases, the average FF and *J*_sc_ are significantly enhanced from 72.8% and 23.5 mA cm^−2^ to 80.3% and 24.6 mA cm^−2^, respectively. The enhanced FF and *J*_sc_ result in a boost in the efficiency from 18.4% to 21.5% on average (20.0 to 23.5% in the champion devices with OAI passivation layer). With 4MEO‐PEAI passivation layer, 24.1% were achieved in the champion device (Figure S10, Supporting Information). A clear tendency with increasing MACl concentration was also shown in the external quantum efficiency (EQE) spectra (Figure 2e). The integrated *J*_sc_ extracted from the EQE spectra increased as the photocurrent generation in the near‐infrared region (nearly up to 850 nm in wavelength) of the spectrum was slightly enhanced. The current density‐voltage characteristics are shown in the *J*–*V* curves in Figure 2f. The shunt and series resistance (*R*_sh_ and *R*_s_, respectively) were extracted from the *J*–*V* curves. Although *R*_sh_ does not show a clear tendency with the MACl concentration, the MACl40% device showed the highest *R*_sh_ of 2267.32 Ω‐cm^2^. *R*_s_, in contrast, showed a clear tendency, as it was gradually reduced from 4.64 Ω‐cm^2^ in MACl10% to 2.81 Ω‐cm^2^ in MACl40%. The enhancement in the FF with the MACl concentration can be explained as follows. As we have already discussed regarding Figure 1 and will be discussed later with AFM characterization, the increased MACl concentration resulted in a (100) preferred crystallographic orientation and an increased grain size with much more homogeneous and flatter. It implies a correlation between the larger grains with the (100) preferred orientation and the enhanced device performance.

**Table 1:** Summary of the photovoltaic parameters of *J*_sc_, *V*_oc_, FF, and PCE of the PSCs with different concentrations of MACl

MACl [%]	*V*_oc_ [V]	*J*_sc_ [mA cm^−2^]	FF [%]	PCE [%]
10 (champion device)	1.08 ± 0.01 (1.11)	23.5 ± 0.25 (23.2)	72.80 ± 1.45 (78.0)	18.40 ± 0.45 (20.0)
20 (champion device)	1.11 ± 0.01 (1.12)	23.9 ± 0.50 (23.8)	76.95 ± 1.81 (79.8)	20.30 ± 0.88 (21.4)
30 (champion device)	1.11 ± 0.01 (1.11)	24.2 ± 0.25 (24.1)	77.75 ± 1.16 (80.5)	20.95 ± 0.38 (21.7)
40 (champion device)	1.08 ± 0.02 (1.12)	24.6 ± 0.31 (25.4)	80.30 ± 1.38 (82.2)	21.49 ± 0.38 (23.5)

### 2.4 Optical Properties of (100)‐Oriented *α*‐FAPbI_3_

**Figure 3:**
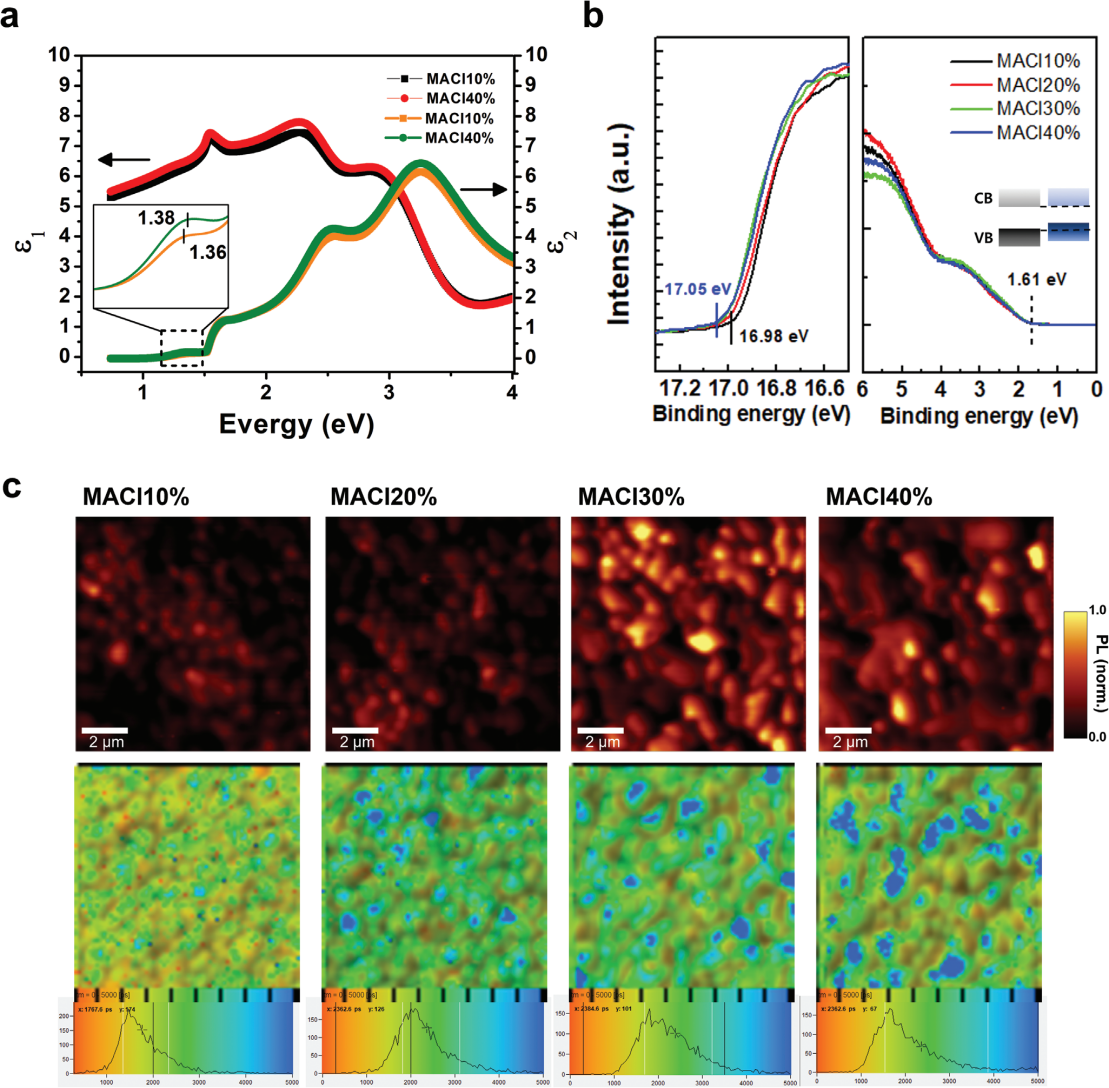
Optical properties of (100)‐oriented *α*‐FAPbI_3_ thin films. a) Dielectric functions *ε*_1_ (real part) and *ε*_2_ (imaginary part) of MACl10% (black and yellow) and MACl40% (red and green) measured by spectroscopic ellipsometry. b) UPS of *α*‐FAPbI_3_ films with 10%, 20%, 30%, 40% MACl. The Fermi level and VBM are extracted from the cutoff edge of UPS spectra marked by solid and vertical lines, respectively. While the CBM of MACl40% is identical, the VBM increases, resulting in the inset schematic band diagram. CB, conduction band; VB, valence band. c) Confocal PL intensity and TRPL mapping of *α*‐FAPbI_3_ films with different concentrations of MACl: 10%, 20%, 30%, 40%. In the PL mapping images, regions with higher PL intensity are observed for both MACl30% and MACl40%. Additionally, in the TRPL mapping images, regions with longer lifetimes (blue) are observed for both MACl30% and MACl40%, as shown in the TRPL distributions. This implies that (100)‐oriented *α*‐FAPbI_3_ thin films suppress nonradiative recombination and increase the PL lifetime compared to randomly oriented *α*‐FAPbI_3_ thin films.

The dispersion of the dielectric constants was measured by spectroscopic ellipsometry, as shown in **Figure** **3**a. The real and imaginary parts of the dielectric constants in both MACl10% and MACl40% thin films showed a slight difference, as MACl40% exhibited fine structures with distinct peak positions in both spectra. Meanwhile, MACl10% exhibits broad peaks with uncertain peak positions. The first peak in the imaginary part of the dielectric function represents a direct optical transition known as the optical gap. The imaginary part of the dielectric function is marked in yellow for MACl10% and green for MACl40% in Figure 3a. The first peaks are located at 1.36 for MACl10% and 1.38 for MACl40%. The redshift of the peak position indicates a slight decrease in the bandgap in MACl40%. This result corresponds to previous studies.^[^ ^83^ ^]^ As the FA cation orientation causes a peak shift in the dielectric functions,^[^ ^84^ ^]^ the randomness of the orientation in MACl10% thin films may induce peak broadening.^[^ ^85^ ^]^ Ultraviolet photoelectron spectroscopy (UPS) was performed to examine the band structures of MACl thin films, as shown in Figure 3b. The Fermi level and valence band maximum (VBM) of the samples were extracted from the UPS data. MACl40% showed a slight upward shift of the VBM (≈70 meV) compared to MACl10%. The conduction band minimum (CBM), in contrast, was identical in both samples. It showed good agreement with previous studies reporting the band energies of the CBM and VBM along different crystallographic directions.^[^ ^86^, ^87^, ^88^, ^89^, ^90^ ^]^ It also suggests that the origin of the optical properties varying with crystallographic orientation, that is, the birefringence, may be due to the band energy difference along the orientation.

Ultraviolet–visible (UV–vis) absorption spectra (Figure S11, Supporting Information) and PL spectra (Figure S12, Supporting Information) of both MACl10% and MACl40% thin films were obtained to compare the optical bandgaps. A slight redshift for MACl40% was observed in both the UV–vis and PL spectra, as the PL peak position shifted from 806 nm (MACl10%) to 812 nm (MACl40%) with an obvious increase in the absorbance. This resulted in a slightly smaller bandgap of MACl40% (≈1.52 eV) compared to MACl10% (≈1.53 eV). This may be induced by the slight upward shift of the VBM in MACl40% (Figure 3b). However, as the upward shift of the VBM (≈70 meV) is larger than the bandgap shortening (≈10 meV), the CBM may also be shifted upward in MACl40%. This can explain why *V*_oc_ did not show any clear tendency with the MACl concentration (Figure 2d). We note that the bandgaps of both MACl10 and 40% extracted from the optical spectroscopies were nearly identical, unrelated to the MACl concentration. This result suggests that adding MACl only stabilizes *α*‐FAPbI_3_ and does not affect the overall intrinsic properties of *α*‐FAPbI_3_.^[^ ^36^, ^91^ ^]^

To demonstrate that the (100) orientation enhances the charge carrier lifetime, we employed confocal PL intensity and TRPL mapping, as shown in Figure 3c. As the MACl concentration increases, regions with higher PL intensity also increase. In the TRPL mapping, regions with longer lifetimes increase with higher MACl concentration. In fact, the TRPL distribution shift toward a longer lifetime indicates preferential spatial homogenization of the TRPL.^[^ ^92^, ^93^ ^]^ With MACl10% representing randomly oriented and MACl40% (100) oriented, this sets up a microscopic view of the PL characteristics of the (100) orientation. As deQuilettes et al. modeled the PL dynamics as a combination of trapping, monomolecular recombination, and bimolecular recombination,^[^ ^94^ ^]^ we measured the trap densities of MACl10% and MACl40% using the space charge limited current (SCLC), as shown in Figure S13, Supporting Information. The calculated trap densities of MACl10% and MACl40% were 3.59 × 10^15^ and 2.57 × 10^15^ cm^−3^, respectively. The lower trap density of MACl40% indicated fewer nonradiative centers, as shown by the confocal PL mappings in Figure 3c.^[^ ^94^, ^95^, ^96^, ^97^ ^]^ The results from Figure 3 suggested that the enhanced optical properties of the (100) orientation originated from the lower trap density of the (100) orientation.^[^ ^98^, ^99^ ^]^ This may explain why MACl40% had a “better shape” of the EQE spectra compared to MACl10%. As EQE spectra govern the maximum *V*_oc_, the solar cell can achieve the “radiative limit.”^[^ ^92^, ^100^ ^]^

### 2.5 Transport Properties of (100)‐Oriented *α*‐FAPbI_3_

**Figure 4:**
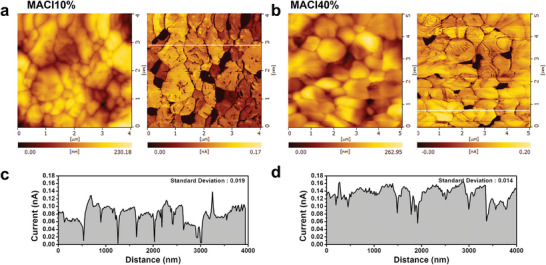
Electrical properties of (100)‐oriented *α*‐FAPbI_3_ thin films. a,b) Surface topography and photocurrent measurement by C‐AFM of MACl10% and MACl40%. Measurements were taken under illumination (green LED) with a 1.3 V bias. Current images indicate that photocurrents were induced grain by grain, as each grain showed a distinct photocurrent. The photocurrent deviation for each grain is larger in MACl10% than in MACl40%, which shows a similar photocurrent grain by grain. c,d) Current line profile extracted from the current image (a and b). The standard deviation calculated from the magnitude of the photocurrent in each grain is displayed in the inset (0.019 and 0.014 nA for MACl10% and MACl40%, respectively). A low photocurrent was exhibited at the grain boundaries in both MACl10% and MACl40%. The dark areas measured within the current images are most likely distributed between grain edges. This may be caused by the formation of PbI_2_ or the loss of contact between the grain and the conducting substrate.

To demonstrate that the (100) orientation enhances the charge carrier transport properties, photocurrent generation was measured by C‐AFM, as shown in **Figure** **4**. The surface topography and corresponding photocurrent images of MACl10% and MACl40% thin films were simultaneously measured under illumination. Using sampling intelligent scan mode, topography, and photocurrent information was gathered at each measurement point. This allowed stable acquisition of topographic images without damaging the sample or reducing the topographic artifacts. In the results, clear images of the photocurrent generated from each grain were obtained (Figure 4a,b). As each grain showed a distinct photocurrent level, the deviation between each grain was larger in MACl10%. This was well verified by the current line profiles in Figure 4c,d. The magnitude of the photocurrent with standard deviations from each grain was obtained. The standard deviation of MACl10% was higher than that of MACl40% (0.019 and 0.014 nA), while the overall photocurrent level in average is higher in MACl40%. The fact that (100)‐oriented MACl40% exhibited much more homogeneous in the photocurrent generation among grains demonstrates the correlation of the crystallographic orientation and charge transport properties.^[^ ^101^, ^102^, ^103^ ^]^ As Leblebici et al. already reported heterogeneity in the drift current due to changes in effective mass, the deviation in the photocurrent can originate from different effective masses along each orientation.^[^ ^102^ ^]^ The surface roughness values of MACl10 and 40% thin films were measured, as shown in Figure S14, Supporting Information. The average roughness (*R*_a_) in areas of the same range is higher in MACl10% (*R*_a_: 17.11 nm) than in MACl40% (*R*_a_: 11.50 nm). This is due to the smaller grain size and rough grain surfaces. A lower *R*_a_ can induce better physical contact between the transport layer and the *α*‐FAPbI_3_ layer, resulting in a higher FF. With the (100) preferentially oriented *α*‐FAPbI_3_ thin film achieving homogeneous generation of a photocurrent along the flat surface, the higher *J*_sc_ and FF of MACl40% compared to randomly oriented MACl10% can be resolved (Figure 2b,c). This eventually contributes to a higher PCE.

### 2.6 Enlarging the Grain Size and Inducing a Preferred Orientation via Two‐Step Heat Treatment

**Figure 5:**
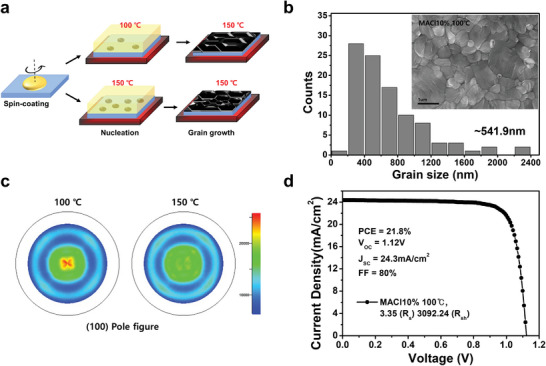
Controlling the grain size and orientation. a) Schematic showing the two types of heat treatment for *α*‐FAPbI_3_ thin films. The one‐step treatment involves holding at a constant temperature of 150 °C for 20 min, while the two‐step treatment involves holding at 100 °C for 10 min and then heating at 150 °C for 10 min. b) The MACl10% thin film produced with the two‐step treatment exhibited a larger grain size, as shown in the SEM histogram. Average grain size (≈541.9 nm) obtained from SEM images set within the histogram. c) (100) pole figures of MACl10% thin films produced by the one‐step (150 °C) and two‐step (100 °C) treatments. The higher intensity of the (100) pole figure demonstrates that MACl10% (100 °C) is (100) oriented. d) *J*–*V* curve of MACl10% (100 °C) thin films. *R*_s_ and *R*_sh_ were extracted from the *J*–*V* curve. PSCs with the structure FTO/ALD‐SnO_2_/SnO_2_ NP/*α*‐FAPbI_3_/OAI/Spiro‐OMeTAD/Au were fabricated.

We have found that the (100) preferred orientation and large grain size led to higher PCEs. To confirm our observations, we successfully improved the PCE of MACl10% by enlarging the grain size and inducing the (100) preferred orientation. In Figure S15, Supporting Information, the Gibbs free energy (*G*_T_) as a function of the nucleus’ radius is depicted. As *G*_T_ consists of both volumetric (*G*_V_) and surface (*G*_S_) terms, the critical radius (*r*_c_) for the start of the nucleation process can be determined. The conventional LaMer's model depicted in Figure S16, Supporting Information also explains the homogeneous nucleation and growth process. LaMer's model shows the concentration of an *α*‐FAPbI_3_ precursor solution as a function of time. As the synthesis of *α*‐FAPbI_3_ thin films requires heat treatment, *α*‐FAPbI_3_ nuclei start to occur when the solution concentration increases and reaches the supersaturation concentration (*C*_s_) (stage I). In stage II, already formed nuclei start to grow while the nucleation continues. In stage III, as the consumption of the solute becomes faster than the evaporation of the solvent, the solution concentration decreases to below *C*_s_, resulting in growth of the nuclei without formation of additional nuclei. Based on both classical homogeneous nucleation theory and LaMer's model, the crystal growth of *α*‐FAPbI_3_ thin films can be controlled by balancing the nucleation and growth processes. By lowering the heat treatment temperature, we were able to suppress nucleation and enhance the growth process. As *G*_v_ strongly depends on the temperature compared to *G*_s_, the overall *G*_t_ decreases as the temperature decreases (Figure S15, Supporting Information). This results in a smaller *r*_c_. Additionally, a lower temperature induces slower evaporation and a lower *C*_s_. Therefore, a smaller *r*_c_ and a lower *C*_s_ result in shorter nucleation and longer growth processes. The schematic in **Figure** **5**a shows two types of heat treatment. We added an additional step of a 100 °C heat treatment to the original one‐step 150 °C treatment, which was used to synthesize *α*‐FAPbI_3_ thin films (MACl10, 20, 30, and 40%). We note that the heat treatment with lower temperature than 150 °C induces slowing down the grain growth. After the grain growth process, temperature was then increased to 150 °C for 10 min to stabilize the *α*‐FAPbI_3_ phase.^[^ ^104^, ^105^ ^]^ With the two‐step heat treatment (100 → 150 °C), we were able to enlarge the grain size of the MACl10% thin film, as shown in Figure 5b. The histogram shows the grain size distribution of ≈100 grains obtained from SEM images. Comparing the average grain size, MACl10% (100 → 150 °C) obtained a larger grain size of ≈540 nm than MACl10% (150 °C), which obtained a grain size of ≈340 nm (Figure S1a, Supporting Information). Moreover, the (100) pole figure in Figure 5c indicates (100)‐oriented growth in the MACl10% (100 → 150 °C) thin film, as the intensity of the (100) pole figure increased compared to that of the MACl10% (150 °C) thin film. This is also examined in the XRD patterns in Figure S17, Supporting Information. As both thin films formed the *α*‐FAPbI_3_ phase, the (100) orientation degree increased in MACl10% (100 → 150 °C). Inducing a longer growth process is demonstrated to result in a larger grain size and a (100) preferred orientation. MACl40% film fabricated by the 2‐step heat treatment was examined by the SEM in Figure S18, Supporting Information. The SEM image shows enlarged average grain size of ≈2,264 nm compared to the 1‐step heat treatment, however, pin holes were examined between the grain boundaries. As the 2‐step heat treatment slower the crystallization kinetic, MACl40% with the 2‐step has induced poor coverage. This indicates that control of the crystallization is important to balance the grain growth and surface coverage.

To investigate whether certain changes in the MACl10% thin film (larger grain size and (100) preferred orientation) affect the device performance, a PSC of MACl10% (100 → 150 °C) was fabricated. The *J*–*V* curve in Figure 5d shows an improved PCE of 21.8% compared to the MACl10% (150 °C) PCE of 18.4% in Table 1. *R*_s_ decreased from 4.64 to 3.35 and *R*_sh_ increased from 904.72 to 3092.24 compared to MACl10% (150 °C) in Figure 2f. An easy comparison of the PV parameters of MACl10% (150 °C) and MACl10% (100 → 150 °C) is shown in Figures S19 and S20, Supporting Information. The massive improvement in the PV parameters (*J*_sc_, FF, and *V*_oc_) was induced by the controlling of crystallization kinetics resulting in the larger grain size and preferred orientation.

## 3 Conclusions

In summary, by adjusting the concentration of MACl additive, we kinetically controlled the crystallization process of *α*‐FAPbI_3_. With higher concentration of MACl resulting nucleation suppression and enhanced growth process, we were able to enlarge the grain size and obtain [100] preferentially oriented growth. As a result, MACl40% film observed the largest average grain size of ≈1 µm and (100) preferred orientation than those with 10, 20, and 30% MACl. Confocal PL and TRPL mapping show higher PL intensity and longer lifetime for MACl40%, indicating that (100)‐oriented grains generate less nonradiative recombination. Meanwhile, C‐AFM measurements indicated that (100) preferentially oriented thin films generate a homogeneous photocurrent. Therefore, with (100) orientation originating higher *J*_sc_ and FF compared to a random orientation, MACl40% obtained the highest PCE of 24.1%. The results indicate that a higher concentration of MACl leads to a high PCE by slowing the crystallization kinetics which induces enlarged grain size and an ordered orientation. To confirm this hypothesis, we controlled the grain size and orientation of the MACl10% thin film by suppressing the nucleation events and increasing the kinetics of grain growth. As the MACl10% thin film synthesized through two‐step heat treatment exhibited an enlarged grain size and a (100) preferred orientation, the overall device performance was improved to 21.8% with the same amount of additives.

A clear understanding of the evolution of microstructure and its related properties in the perovskite absorbers are crucial, as it provides us with a fundamental perspective toward future developments in highly efficient PSCs. Yet not much progress has been made on studies of controlling the microstructural evolution through the crystallization process. In this study, we suggest the kinetic control of perovskite crystallization process can significantly influence the microstructural evolution, furthermore, materials’ design for the enhanced device performance.

## 4 Experimental Section

Formamidinium iodide (CH5NH2I, FAI) was purchased from Dyesol Ltd. (Queanbeyan, Australia). 2,2“,7,7”‐Tetrakis[*N*,*N*‐di(4‐methoxyphenyl)amino]‐9,9′‐spirobifluorene (Spiro‐OMeTAD) was purchased from Luminescence Technology Corp. (Taiwan). Lead(II) iodide (PbI2, 99.999%) and methylammonium chloride (CH3NH3Cl, MACl) were purchased from Alfa Aesar. Anhydrous dimethylformamide (DMF, 99.9%), anhydrous dimethylsulfoxide (DMSO, 99.9%), anhydrous chlorobenzene (99.8%), anhydrous 2‐methoxyethanol (2‐ME, 99.8%), anhydrous 2‐propanol (IPA, 99.5%), *n*‐octylammonium iodide, and 4‐methoxy‐phenethylammonium iodide were purchased from Sigma Aldrich Co., Ltd. (St Louis, MO, USA). All other chemicals were purchased from Sigma Aldrich or Alfa Aesar unless otherwise specified.

### Synthesis of FAPbI_3_ Powder

Formamidinium lead triiodide (FAPbI_3_) was synthesized using a 1 m solution of PbI_2_ and FAI at an equal molar ratio in 10 mL of 2‐methoxyethanol. FAPbI_3_ powder was obtained by precipitation in 150 °C heated anhydrous anisole. The powder was then filtered and heated to 150 °C to evaporate the remaining solvents.

Nuclear magnetic resonance (NMR, AVANCE III 700, Bruker) spectroscopy was used to obtain the composition of FAI and MAI within the MACl‐added FAPbI_3_. XPS and UPS (ESCALAB 250, ThermoFisher Scientific Inc.) were used to obtain the elemental composition, work function, and VBM.

### Structural Characterizations

Field emission scanning electron microscopy (FESEM, JSM7600F, JEOL) was used to observe the morphology of FAPbI_3_ thin films. X‐ray diffraction (XRD) patterns and pole figures (SmartLab, Rikaku) were used to interpret the crystal structures and crystallographic orientation of FAPbI_3_ thin films. Synchrotron‐based grazing‐incidence XRD (GIXRD) measurements were conducted at the 9A U‐SAXS beamline of the Pohang Light Source in South Korea. The wavelength of the X‐rays was 1.12370 A (*E* = 11.035 keV), and the incidence angle of the light beam ranged from 0.1° to 0.4°. EBSD (EDAX) and the NPAR program (OIM analysis, EDAX) were used to obtain crystallographic information of each grain. It was noted that ion beam milling (5 kV for 30 s) was used before performing EBSD to reduce the surface roughness to obtain clear EBSD images. The crystal structure of each grain was observed by cross‐section transmission electron microscopy (TEM, JEM ARM 200F, JEOL), while a focused ion beam (FIB, JIB‐4601F, JEOL) was used for TEM sample preparation.

### Crystallization Kinetic Characterization

Isothermal crystallization process of FAPbI_3_ solutions was recorded by using optical microscope (BH2‐UMA, OLYMPUS) with an objective lens, IC20 (objective magnification, 20×). The solutions were dropped onto the substrates and then were spin coated at 6000 rpm for 15 s before transferring to a hot plate of 100 °C. Considering the crystal growth to be a 2‐demensional growth, fractional crystallinity curves were obtained by carefully observing the growth of the crystal over a specific area of (2000 um × 1500 um). Crystallization rates were calculated by the Avrami plot of fractional crystallization. With the Avrami model, fraction of transformed material (*Y*) after a certain time (*t*) at a constant temperature, Avrami equation can be calculated by the following form.

(1) $$Y\;=\;1-\exp\left(-Z_{t}t^{n}\right)$$

The equation can be transformed by taking logarithm on both sides.

(2) $$\ln\left[-\ln\left(1-X\left(t\right)\right)\right]=\;n\ln t+\ln Z_{t}$$

*Z*_t_ and *n* represent the kinetic rate constant and Avrami exponent, respectively. With ln [ − ln (1 − *X*(*t*))] plotted against ln *t*, the intercept of the graph is *Z*_t_ and the slope is *n*.

### Device Fabrication

Fluorine‐doped tin oxide (FTO) glass (Asahi VU glass) with dimensions of 21 × 21 mm was patterned by wet etching (etched area: 4 × 21 mm) with Zn powder and a 4 m aqueous solution of HCl. The substrates were then cleaned in an ultrasonic bath in a series of detergent solutions (2 wt% Hellmanex III in deionized (DI) water), DI water, acetone, and ethanol for 10 min each. FTO substrates were then treated in a UV/ozone cleaner for 20 min and used for SnO_2_ deposition via atomic layer deposition (ALD). On top of the ALD‐grown SnO_2_ film, SnO_2_ nanoparticles dispersed in DI water (2%) were spin coated at 4000 rpm for 30 s. The films were annealed at 180 °C for 1 h. The substrates were further UV‐O_3_ treated for 20 min before perovskite deposition.

A perovskite precursor solution (1.6 m FAPbI_3_) was prepared in a mixture of DMF and DMSO (4:1 v/v) solvents. MACl was added to the solution at concentrations of 10, 20, 30, and 40 mol%. The solution (25 µL) was dropped onto the substrate and then spin coated at 6000 rpm for 40 s. During spinning, diethyl ether (1 mL) was spread for solvent engineering and transferred to a hot plate at 150 °C for 10 min. After cooling to room temperature, a 15 mm solution of OAI or 16 mm solution of 4MEO‐PEAI (in IPA) was spin coated at 3000 rpm and 5000 rpm respectively, for 30 s and then heated to 100 °C for 5 min. After cooling, the Spiro‐OMeTAD solution was spread on top of the film at 4000 rpm for 30 s. The Spiro‐OMeTAD solution was prepared with the following composition: 30 mg of Spiro‐OMeTAD, 12.7 µL of 4‐tert‐butylpyridine, 7.7 µL of Li‐TFSI (520 mg mL^−1^ in acetonitrile), and Co‐TFSI (375 mg mL^−1^ in acetonitrile) in 300 µL of chlorobenzene. Au (80 nm thickness) was thermally deposited to complete the device.

### Device Characterization

*J*–*V* measurements were performed using a Keithley 2400 source meter under simulated AM 1.5G one sun illumination (100 mW cm^−2^) using a solar simulator (Oriel Sol 3A class AAA) equipped with a 1600 W xenon lamp (Newport 94083A) and calibrated using an NREL‐calibrated Si reference cell with a KG‐5 filter (PV measurements). Performance validation of the spectral match was within the acceptable limits of Class A spectral match (mismatch to all intervals, 0.75–1.25). *J*–*V* characteristic were measured in the reverse scan direction at a scan rate of 167 mV s^−1^ (150 points with the dwell time of 50 ms). The illuminated active area was fixed by a metal shadow mask (0.06 cm^2^) during the measurement. The external quantum efficiency (EQE) was measured using a QuantX‐300 Quantum Efficiency System (Newport), where a monochromatic beam was generated by chopping the white light using a 100 W xenon lamp. All of the *J*–*V* and EQE measurements were conducted in a bare air condition at room temperature. For each sample, 35 cells were used to collect the statistical PV parameters.

### Optical Characterizations

Photoluminescence (PL) intensity spectra were recorded with a fluorescence lifetime spectrometer (C11367, Hamamatsu Photonics K.K.). UV–vis spectroscopy (OPTIZEN POP, Mecasys) was used to obtain transmittance and absorption spectra of FAPbI_3_ films. A spectroscopic ellipsometer (UVISEL PLUS, Horiba) was used to obtain the dielectric function of FAPbI_3_ films with an incident angle of 60°. Confocal PL and time‐resolved PL mapping were conducted using near‐field optical microscopy (NSOM, Alpha‐300S, WITec Instrument GmbH).

### Electrical Characterizations

AFM and conductive‐AFM (C‐AFM, Nanonavi II, SII Nanotechnology) were used to measure the local conductance of FAPbI_3_ films. All measurements were performed under illumination (green LED) with a 1.3 V bias using a Pt‐coated C‐AFM probe (ContPt, Nanoworld). FTO was used for the conductive substrates.

## Conflict of Interest

The authors declare no conflict of interest.

## Supporting information

Supporting Information

Supporting Information

## Data Availability

Research data are not shared.

## References

[1] L. Gránásy , T. Pusztai , T. Börzsönyi , J. A. Warren , J. F. Douglas , Nat. Mater. 2004, 3, 645. 15300243 10.1038/nmat1190

[2] Q. Gao , J. Ai , S. Tang , M. Li , Y. Chen , J. Huang , H. Tong , L. Xu , L. Xu , H. Tanaka , P. Tan , Nat. Mater. 2021, 20, 1431. 33958770 10.1038/s41563-021-00993-6

[3] S. W. Schaffter , D. Scalise , T. M. Murphy , A. Patel , R. Schulman , Nat. Commun. 2020, 11, 6057. 33247122 10.1038/s41467-020-19882-8 PMC7695852

[4] B. C. Park , J. Cho , M. S. Kim , M. J. Ko , L. Pan , J. Y. Na , Y. K. Kim , Nat. Commun. 2020, 11, 298. 31941908 10.1038/s41467-019-14168-0 PMC6962372

[5] Z. Wang , K. Gao , Y. Kan , M. Zhang , C. Qiu , L. Zhu , Z. Zhao , X. Peng , W. Feng , Z. Qian , X. Gu , A. K. Jen , B. Z. Tang , Y. Cao , Y. Zhang , F. Liu , Nat. Commun. 2021, 12, 332. 33436619 10.1038/s41467-020-20515-3 PMC7804468

[6] M. Hansen , (1958), Constitution of Binary Alloys, 2nd Edition, McGraw‐Hill.

[7] C. Xu , Z. Zhang , S. Zhang , H. Si , S. Ma , W. Fan , Z. Xiong , Q. Liao , A. Sattar , Z. Kang , Y. Zhang , Adv. Funct. Mater. 2021, 31, 2009425.

[8] R. Quintero‐Bermudez , A. Gold‐Parker , A. H. Proppe , R. Munir , Z. Yang , S. O. Kelley , A. Amassian , M. F. Toney , E. H. Sargent , Nat. Mater. 2018, 17, 900. 30202112 10.1038/s41563-018-0154-x

[9] S. Pratap , F. Babbe , N. S. Barchi , Z. Yuan , T. Luong , Z. Haber , T. B. Song , J. L. Slack , C. V. Stan , N. Tamura , C. M. Sutter‐Fella , P. Muller‐Buschbaum , Nat. Commun. 2021, 12, 5624. 34561460 10.1038/s41467-021-25898-5 PMC8463609

[10] D. T. Moore , H. Sai , K. W. Tan , D. M. Smilgies , W. Zhang , H. J. Snaith , U. Wiesner , L. A. Estroff , J. Am. Chem. Soc. 2015, 137, 2350. 25625616 10.1021/ja512117e

[11] N. Zhou , Y. Shen , L. Li , S. Tan , N. Liu , G. Zheng , Q. Chen , H. Zhou , J. Am. Chem. Soc. 2018, 140, 459. 29243924 10.1021/jacs.7b11157

[12] L. Q. Xie , L. Chen , Z. A. Nan , H. X. Lin , T. Wang , D. P. Zhan , J. W. Yan , B. W. Mao , Z. Q. Tian , J. Am. Chem. Soc. 2017, 139, 3320. 28211690 10.1021/jacs.6b12432

[13] H. Min , D. Y. Lee , J. Kim , G. Kim , K. S. Lee , J. Kim , M. J. Paik , Y. K. Kim , K. S. Kim , M. G. Kim , T. J. Shin , S. Il Seok , Nature 2021, 598, 444. 34671136 10.1038/s41586-021-03964-8

[14] A. Y. Alsalloum , B. Turedi , X. Zheng , S. Mitra , A. A. Zhumekenov , K. J. Lee , P. Maity , I. Gereige , A. AlSaggaf , I. S. Roqan , O. F. Mohammed , O. M. Bakr , ACS Energy Lett. 2020, 5, 657.

[15] Y. Li , J. Shi , J. Zheng , J. Bing , J. Yuan , Y. Cho , S. Tang , M. Zhang , Y. Yao , C. F. J. Lau , D. S. Lee , C. Liao , M. A. Green , S. Huang , W. Ma , A. W. Y. Ho‐Baillie , Adv. Sci. 2020, 7, 1903368. 10.1002/advs.201903368 PMC7055551 32154088

[16] Y. Yun , F. Wang , H. Huang , Y. Fang , S. Liu , W. Huang , Z. Cheng , Y. Liu , Y. Cao , M. Gao , L. Zhu , L. Wang , T. Qin , W. Huang , Adv. Mater. 2020, 32, 1907123. 10.1002/adma.201907123 32083790

[17] M. Saliba , T. Matsui , J. Y. Seo , K. Domanski , J. P. Correa‐Baena , M. K. Nazeeruddin , S. M. Zakeeruddin , W. Tress , A. Abate , A. Hagfeldt , M. Gratzel , Energy Environ. Sci. 2016, 9, 1989. 27478500 10.1039/c5ee03874j PMC4936376

[18] R. Wang , J. Xue , K.‐L. Wang , Z.‐K. Wang , Y. Luo , D. Fenning , G. Xu , S. Nuryyeva , T. Huang , Y. Zhao , J. L. Yang , J. Zhu , M. Wang , S. Tan , I. Yavuz , K. N. Houk , Y. Yang , Science 2019, 366, 1509. 31857483 10.1126/science.aay9698

[19] X. Zheng , Y. Hou , C. Bao , J. Yin , F. Yuan , Z. Huang , K. Song , J. Liu , J. Troughton , N. Gasparini , C. Zhou , Y. Lin , D.‐J. Xue , B. Chen , A. K. Johnston , N. Wei , M. N. Hedhili , M. Wei , A. Y. Alsalloum , P. Maity , B. Turedi , C. Yang , D. Baran , T. D. Anthopoulos , Y. Han , Z.‐H. Lu , O. F. Mohammed , F. Gao , E. H. Sargent , O. M. Bakr , Nat. Energy 2020, 5, 131.

[20] J. J. Yoo , G. Seo , M. R. Chua , T. G. Park , Y. Lu , F. Rotermund , Y. K. Kim , C. S. Moon , N. J. Jeon , J. P. Correa‐Baena , V. Bulovic , S. S. Shin , M. G. Bawendi , J. Seo , Nature 2021, 590, 587. 33627807 10.1038/s41586-021-03285-w

[21] J. Jeong , M. Kim , J. Seo , H. Lu , P. Ahlawat , A. Mishra , Y. Yang , M. A. Hope , F. T. Eickemeyer , M. Kim , Y. J. Yoon , I. W. Choi , B. P. Darwich , S. J. Choi , Y. Jo , J. H. Lee , B. Walker , S. M. Zakeeruddin , L. Emsley , U. Rothlisberger , A. Hagfeldt , D. S. Kim , M. Gratzel , J. Y. Kim , Nature 2021, 592, 381. 33820983 10.1038/s41586-021-03406-5

[22] J. W. Lee , S. Seo , P. Nandi , H. S. Jung , N. G. Park , H. Shin , iScience 2021, 24, 101959. 33437939 10.1016/j.isci.2020.101959 PMC7788097

[23] Q. Han , S. H. Bae , P. Sun , Y. T. Hsieh , Y. M. Yang , Y. S. Rim , H. Zhao , Q. Chen , W. Shi , G. Li , Y. Yang , Adv. Mater. 2016, 28, 2253. 26790006 10.1002/adma.201505002

[24] N. J. Jeon , H. Na , E. H. Jung , T.‐Y. Yang , Y. G. Lee , G. Kim , H.‐W. Shin , S. Il Seok , J. Lee , J. Seo , Nat. Energy 2018, 3, 682.

[25] J. W. Lee , S. G. Kim , S. H. Bae , D. K. Lee , O. Lin , Y. Yang , N. G. Park , Nano Lett. 2017, 17, 4270. 28586229 10.1021/acs.nanolett.7b01211

[26] P. Nandi , Z. Li , Y. Kim , T. K. Ahn , N.‐G. Park , H. Shin , ACS Energy Lett. 2021, 6, 837.

[27] D. Y. Son , J. W. Lee , Y. J. Choi , I. H. Jang , S. Lee , P. J. Yoo , H. Shin , N. Ahn , M. Choi , D. Kim , N. G. Park , Nat. Energy 2016, 1, 16081.

[28] M. Kulbak , D. Cahen , G. Hodes , J. Phys. Chem. Lett. 2015, 6, 2452. 26266718 10.1021/acs.jpclett.5b00968

[29] H. Choi , J. Jeong , H.‐B. Kim , S. Kim , B. Walker , G.‐H. Kim , J. Y. Kim , Nano Energy 2014, 7, 80.

[30] J.‐W. Lee , D.‐H. Kim , H.‐S. Kim , S.‐W. Seo , S. M. Cho , N.‐G. Park , Adv. Energy Mater. 2015, 5, 1501310.

[31] Y. Zhang , S. Seo , S. Y. Lim , Y. Kim , S.‐G. Kim , D.‐K. Lee , S.‐H. Lee , H. Shin , H. Cheong , N.‐G. Park , ACS Energy Lett. 2019, 5, 360.

[32] M. Mateen , Z. Arain , Y. Yang , X. Liu , S. Ma , C. Liu , Y. Ding , X. Ding , M. Cai , S. Dai , ACS Appl. Mater. Interfaces 2020, 12, 10535. 32046480 10.1021/acsami.9b22719

[33] T. Zhu , D. Zheng , M.‐N. Rager , T. Pauporté , Sol. RRL 2020, 4, 2000348.

[34] F. Xie , C.‐C. Chen , Y. Wu , X. Li , M. Cai , X. Liu , X. Yang , L. Han , Energy Environ. Sci. 2017, 10, 1942.

[35] M. Kim , G.‐H. Kim , T. K. Lee , I. W. Choi , H. W. Choi , Y. Jo , Y. J. Yoon , J. W. Kim , J. Lee , D. Huh , H. Lee , S. K. Kwak , J. Y. Kim , D. S. Kim , Joule 2019, 3, 2179.

[36] Z. Wang , Y. Zhou , S. Pang , Z. Xiao , J. Zhang , W. Chai , H. Xu , Z. Liu , N. P. Padture , G. Cui , Chem. Mater. 2015, 27, 7149.

[37] J. Qing , X.‐K. Liu , M. Li , F. Liu , Z. Yuan , E. Tiukalova , Z. Yan , M. Duchamp , S. Chen , Y. Wang , S. Bai , J.‐M. Liu , H. J. Snaith , C.‐S. Lee , T. C. Sum , F. Gao , Adv. Energy Mater. 2018, 8, 1800185.

[38] Z. Xu , Z. Liu , N. Li , G. Tang , G. Zheng , C. Zhu , Y. Chen , L. Wang , Y. Huang , L. Li , N. Zhou , J. Hong , Q. Chen , H. Zhou , Adv. Mater. 2019, 31, 1900390. 10.1002/adma.201900390 31012204

[39] A. Z. Chen , B. J. Foley , J. H. Ma , M. R. Alpert , J. S. Niezgoda , J. J. Choi , J. Mater. Chem. A 2017, 5, 7796.

[40] C. Fei , L. Guo , B. Li , R. Zhang , H. Fu , J. Tian , G. Cao , Nano Energy 2016, 27, 17.

[41] E. L. Unger , A. R. Bowring , C. J. Tassone , V. L. Pool , A. Gold‐Parker , R. Cheacharoen , K. H. Stone , E. T. Hoke , M. F. Toney , M. D. McGehee , Chem. Mater. 2014, 26, 7158.

[42] M. Lyu , N.‐G. Park , Sol. RRL 2020, 4, 2000331.

[43] M. Kim , T. K. Lee , I. W. Choi , H. W. Choi , Y. Jo , J. Lee , G.‐H. Kim , S. K. Kwak , D. S. Kim , Sustainable Energy Fuels 2020, 4, 3753.

[44] Y. Wu , X. Li , S. Fu , L. Wan , J. Fang , J. Mater. Chem. A 2019, 7, 8078.

[45] R. D. Chavan , D. Prochowicz , P. Yadav , M. M. Tavakoli , A. Nimbalkar , S. P. Bhoite , C. K. Hong , Sol. RRL 2019, 3, 1900294.

[46] H. T. Pham , Y. Yin , G. Andersson , K. J. Weber , T. Duong , J. Wong‐Leung , Nano Energy 2021, 87, 106226.

[47] F. Zhang , J. Cong , Y. Li , J. Bergstrand , H. Liu , B. Cai , A. Hajian , Z. Yao , L. Wang , Y. Hao , X. Yang , J. M. Gardner , H. Ågren , J. Widengren , L. Kloo , L. Sun , Nano Energy 2018, 53, 405.

[48] F. Wen , L. Tian , W. Zhang , P. Lin , X. Zhou , S. Zhou , D. Huang , J. He , X. Shen , C. Peng , Z. Ma , Y. Huang , J. Phys. Chem. C 2021, 125, 19131.

[49] J. Chen , J. Song , F. Huang , H. Li , S. Liu , M. Wang , Y. Shen , J. Phys. Chem. C 2017, 121, 17053.

[50] B. Chen , Z. Yu , K. Liu , X. Zheng , Y. Liu , J. Shi , D. Spronk , P. N. Rudd , Z. Holman , J. Huang , Joule 2019, 3, 177.

[51] B. Lee , T. Hwang , S. Lee , B. Shin , B. Park , Sci. Rep. 2019, 9, 4803. 30886329 10.1038/s41598-019-41328-5 PMC6423327

[52] C. Liu , Y. B. Cheng , Z. Ge , Chem. Soc. Rev. 2020, 49, 1653. 32134426 10.1039/c9cs00711c

[53] J. H. Im , I. H. Jang , N. Pellet , M. Gratzel , N. G. Park , Nat. Nanotechnol. 2014, 9, 927. 25173829 10.1038/nnano.2014.181

[54] S. Paek , P. Schouwink , E. N. Athanasopoulou , K. T. Cho , G. Grancini , Y. Lee , Y. Zhang , F. Stellacci , M. K. Nazeeruddin , P. Gao , Chem. Mater. 2017, 29, 3490.

[55] Y. Wang , J. Wu , P. Zhang , D. Liu , T. Zhang , L. Ji , X. Gu , Z. D. Chen , S. Li , Nano Energy 2017, 39, 616.

[56] M. Xiao , F. Huang , W. Huang , Y. Dkhissi , Y. Zhu , J. Etheridge , A. Gray‐Weale , U. Bach , Y. B. Cheng , L. Spiccia , Angew. Chem., Int. Ed. 2014, 53, 9898. 10.1002/anie.201405334 25047967

[57] F. Huang , Y. Dkhissi , W. Huang , M. Xiao , I. Benesperi , S. Rubanov , Y. Zhu , X. Lin , L. Jiang , Y. Zhou , A. Gray‐Weale , J. Etheridge , C. R. McNeill , R. A. Caruso , U. Bach , L. Spiccia , Y.‐B. Cheng , Nano Energy 2014, 10, 10.

[58] M. Kim , G. H. Kim , K. S. Oh , Y. Jo , H. Yoon , K. H. Kim , H. Lee , J. Y. Kim , D. S. Kim , ACS Nano 2017, 11, 6057. 28505416 10.1021/acsnano.7b02015

[59] J. W. Lee , Z. Dai , C. Lee , H. M. Lee , T. H. Han , N. De Marco , O. Lin , C. S. Choi , B. Dunn , J. Koh , D. Di Carlo , J. H. Ko , H. D. Maynard , Y. Yang , J. Am. Chem. Soc. 2018, 140, 6317. 29723475 10.1021/jacs.8b01037

[60] N. Ahn , D. Y. Son , I. H. Jang , S. M. Kang , M. Choi , N. G. Park , J. Am. Chem. Soc. 2015, 137, 8696. 26125203 10.1021/jacs.5b04930

[61] P. W. Liang , C. Y. Liao , C. C. Chueh , F. Zuo , S. T. Williams , X. K. Xin , J. Lin , A. K. Jen , Adv. Mater. 2014, 26, 3748. 24634141 10.1002/adma.201400231

[62] G. E. Eperon , S. D. Stranks , C. Menelaou , M. B. Johnston , L. M. Herz , H. J. Snaith , Energy Environ. Sci. 2014, 7, 982.

[63] D. P. McMeekin , Z. Wang , W. Rehman , F. Pulvirenti , J. B. Patel , N. K. Noel , M. B. Johnston , S. R. Marder , L. M. Herz , H. J. Snaith , Adv. Mater. 2017, 29, 1607039. 10.1002/adma.201607039 28561912

[64] C. Ji , C. Liang , H. Zhang , M. Sun , Q. Song , F. Sun , X. Feng , N. Liu , H. Gong , D. Li , F. You , Z. He , ACS Appl. Mater. Interfaces 2020, 12, 20026. 32249563 10.1021/acsami.9b23468

[65] H. Fan , J.‐H. Huang , L. Chen , Y. Zhang , Y. Wang , C. Gao , P. Wang , X. Zhou , K.‐J. Jiang , Y. Song , J. Mater. Chem. A 2021, 9, 7625.

[66] Z. Xiao , Q. Dong , C. Bi , Y. Shao , Y. Yuan , J. Huang , Adv. Mater. 2014, 26, 6503. 25158905 10.1002/adma.201401685

[67] S. Xiao , Y. Bai , X. Meng , T. Zhang , H. Chen , X. Zheng , C. Hu , Y. Qu , S. Yang , Adv. Funct. Mater. 2017, 27, 1604944.

[68] S. Bag , M. F. Durstock , ACS Appl. Mater. Interfaces 2016, 8, 5053. 26862869 10.1021/acsami.5b11494

[69] M. M. Tavakoli , P. Yadav , D. Prochowicz , M. Sponseller , A. Osherov , V. Bulović , J. Kong , Adv. Energy Mater. 2019, 9, 1803587.

[70] V. Murugan , Y. Ogomi , S. S. Pandey , T. Toyoda , Q. Shen , S. Hayase , Appl. Phys. Express 2015, 8, 125501.

[71] H. Yu , F. Wang , F. Xie , W. Li , J. Chen , N. Zhao , Adv. Funct. Mater. 2014, 24, 7102.

[72] S. Jariwala , H. Sun , G. W. P. Adhyaksa , A. Lof , L. A. Muscarella , B. Ehrler , E. C. Garnett , D. S. Ginger , Joule 2019, 3, 3048.

[73] T. Leonhard , A. D. Schulz , H. Röhm , S. Wagner , F. J. Altermann , W. Rheinheimer , M. J. Hoffmann , A. Colsmann , Energy Technol. 2019, 7, 1800989.

[74] G. W. P. Adhyaksa , S. Brittman , H. Abolins , A. Lof , X. Li , J. D. Keelor , Y. Luo , T. Duevski , R. M. A. Heeren , S. R. Ellis , D. P. Fenning , E. C. Garnett , Adv. Mater. 2018, 30, 1804792. 10.1002/adma.201804792 30368936

[75] B.‐w. Park , H. W. Kwon , Y. Lee , D. Y. Lee , M. G. Kim , G. Kim , K.‐j. Kim , Y. K. Kim , J. Im , T. J. Shin , S. I. Seok , Nat. Energy 2021, 6, 419.

[76] M. I. Dar , N. Arora , P. Gao , S. Ahmad , M. Gratzel , M. K. Nazeeruddin , Nano Lett. 2014, 14, 6991. 25392941 10.1021/nl503279x

[77] G. Grancini , S. Marras , M. Prato , C. Giannini , C. Quarti , F. De Angelis , M. De Bastiani , G. E. Eperon , H. J. Snaith , L. Manna , A. Petrozza , J. Phys. Chem. Lett. 2014, 5, 3836. 26278757 10.1021/jz501877h

[78] C. Ma , M.‐C. Kang , S.‐H. Lee , S. J. Kwon , H.‐W. Cha , C.‐W. Yang , N.‐G. Park , Joule 2022, 6, 2626.

[79] S. Seo , S. Jeong , H. Park , H. Shin , N. G. Park , Chem. Commun. 2019, 55, 2403. 10.1039/c8cc09578g 30719523

[80] S. Jeong , S. Seo , H. Park , H. Shin , Chem. Commun. 2019, 55, 2433. 10.1039/c8cc09557d 30687861

[81] S. Jeong , S. Seo , H. Yang , H. Park , S. Shin , H. Ahn , D. Lee , J. H. Park , N. G. Park , H. Shin , Adv. Energy Mater. 2021, 11, 2102236.

[82] S. Seo , S. Shin , E. Kim , S. Jeong , N.‐G. Park , H. Shin , ACS Energy Lett. 2021, 6, 3332.

[83] A. Mehdizadeh , S. F. Akhtarianfar , S. Shojaei , J. Phys. Chem. C 2019, 123, 6725.

[84] S. Liu , J. Wang , Z. Hu , Z. Duan , H. Zhang , W. Zhang , R. Guo , F. Xie , Sci. Rep. 2021, 11, 20433. 34650139 10.1038/s41598-021-99621-1 PMC8517011

[85] S. Funke , B. Miller , E. Parzinger , P. Thiesen , A. W. Holleitner , U. Wurstbauer , J. Phys.: Condens. Matter 2016, 28, 385301. 27460278 10.1088/0953-8984/28/38/385301

[86] C. Quarti , C. Katan , J. Even , J. Phys. Mater. 2020, 3, 042001.

[87] P. Zhao , J. Su , Z. Lin , J. Wang , J. Zhang , Y. Hao , X. Ouyang , J. Chang , Mater. Today Energy 2020, 17, 100481.

[88] M. Kato , T. Fujiseki , T. Miyadera , T. Sugita , S. Fujimoto , M. Tamakoshi , M. Chikamatsu , H. Fujiwara , J. Appl. Phys. 2017, 121, 115501.

[89] Y. Wang , Y. Zhang , P. Zhang , W. Zhang , Phys. Chem. Chem. Phys. 2015, 17, 11516. 25855411 10.1039/c5cp00448a

[90] C. Quarti , F. De Angelis , D. Beljonne , Chem. Mater. 2017, 29, 958.

[91] M. K. Hanul Min , S.‐U. Lee , H. Kim , G. Kim , K. Choi , J. H. Lee , S. I. Seok , Science 2019, 366, 749. 31699938 10.1126/science.aay7044

[92] J. T.‐W. Wang , Z. Wang , S. Pathak , W. Zhang , D. W. deQuilettes , F. Wisnivesky‐Rocca‐Rivarola , J. Huang , P. K. Nayak , J. B. Patel , H. A. Mohd Yusof , Y. Vaynzof , R. Zhu , I. Ramirez , J. Zhang , C. Ducati , C. Grovenor , M. B. Johnston , D. S. Ginger , R. J. Nicholas , H. J. Snaith , Energy Environ. Sci. 2016, 9, 2892.

[93] J. Haruyama , K. Sodeyama , L. Han , Y. Tateyama , J. Phys. Chem. Lett. 2014, 5, 2903. 26278097 10.1021/jz501510v

[94] D. W. deQuilettes , S. M. Vorpahl , S. D. Stranks , H. Nagaoka , G. E. Eperon , M. E. Ziffer , H. J. Snaith , D. S. Ginger , Science 2015, 348, 683. 25931446 10.1126/science.aaa5333

[95] M. Abdi‐Jalebi , Z. Andaji‐Garmaroudi , S. Cacovich , C. Stavrakas , B. Philippe , J. M. Richter , M. Alsari , E. P. Booker , E. M. Hutter , A. J. Pearson , S. Lilliu , T. J. Savenije , H. Rensmo , G. Divitini , C. Ducati , R. H. Friend , S. D. Stranks , Nature 2018, 555, 497. 29565365 10.1038/nature25989

[96] G. Xing , N. Mathews , S. S. Lim , N. Yantara , X. Liu , D. Sabba , M. Gratzel , S. Mhaisalkar , T. C. Sum , Nat. Mater. 2014, 13, 476. 24633346 10.1038/nmat3911

[97] S. D. Stranks , V. M. Burlakov , T. Leijtens , J. M. Ball , A. Goriely , H. J. Snaith , Phys. Rev. Appl. 2014, 2, 034007.

[98] H. Uratani , K. Yamashita , J. Phys. Chem. Lett. 2017, 8, 742. 28129504 10.1021/acs.jpclett.7b00055

[99] B. J. Foley , S. Cuthriell , S. Yazdi , A. Z. Chen , S. M. Guthrie , X. Deng , G. Giri , S. H. Lee , K. Xiao , B. Doughty , Y. Z. Ma , J. J. Choi , Nano Lett. 2018, 18, 6271. 30216078 10.1021/acs.nanolett.8b02417

[100] K. Tvingstedt , O. Malinkiewicz , A. Baumann , C. Deibel , H. J. Snaith , V. Dyakonov , H. J. Bolink , Sci. Rep. 2014, 4, 6071. 25317958 10.1038/srep06071 PMC5377528

[101] C. Jiang , P. Zhang , J. Appl. Phys. 2018, 123, 083105.

[102] S. Y. Leblebici , L. Leppert , Y. Li , S. E. Reyes‐Lillo , S. Wickenburg , E. Wong , J. Lee , M. Melli , D. Ziegler , D. K. Angell , D. F. Ogletree , P. D. Ashby , F. M. Toma , J. B. Neaton , I. D. Sharp , A. Weber‐Bargioni , Nat. Energy 2016, 1, 16093.

[103] G. Zheng , C. Zhu , J. Ma , X. Zhang , G. Tang , R. Li , Y. Chen , L. Li , J. Hu , J. Hong , Q. Chen , X. Gao , H. Zhou , Nat. Commun. 2018, 9, 2793. 30022027 10.1038/s41467-018-05076-w PMC6052040

[104] B. Kim , J. Kim , N. Park , Sci. Rep. 2020, 10, 19635. 33184384 10.1038/s41598-020-76742-7 PMC7665211

[105] V. L. Pool , B. Dou , D. G. Van Campen , T. R. Klein‐Stockert , F. S. Barnes , S. E. Shaheen , M. I. Ahmad , M. F. van Hest , M. F. Toney , Nat. Commun. 2017, 8, 14075. 28094249 10.1038/ncomms14075 PMC5247577

