# Supplementary material for: Kinetic‐Controlled Crystallization of α‐FAPbI3 Inducing Preferred Crystallographic Orientation Enhances Photovoltaic Performance
Source: Adv Sci (Weinh). 2023 Mar 30;10(14):2300798. doi: 10.1002/advs.202300798 (PMC10190499; doi:10.1002/advs.202300798)
Supplement: Supplementary file 1 — Supporting Information [file ADVS-10-2300798-s001.pdf]

ADVANCED SCIENCE
Open Access

Supporting Information

for *Adv. Sci.*, DOI 10.1002/advs.202300798

Kinetic-Controlled Crystallization of α-$FAPbI_3$ Inducing Preferred Crystallographic Orientation Enhances Photovoltaic Performance

Sooeun Shin, Seongrok Seo, Seonghwa Jeong, Anir S. Sharbirin, Jeongyong Kim, Hyungju Ahn, Nam-Gyu Park and Hyunjung Shin**

Kinetic-controlled crystallization of α-$FAPbI_3$ inducing preferred crystallographic orientation enhances photovoltaic performance

Sooeun Shin[1,2], Seongrok Seo[3], Seonghwa Jeong[1], Anir S. Sharbirin[1], Jeongyong Kim[1], Hyungju Ahn[4], Nam-Gyu Park[2,5,] and Hyunjung Shin[1,2,*]*

[1]Department of Energy Science, Sungkyunkwan University, Suwon 440-746, Republic of Korea

[2]SKKU Institute of Energy Science and Technology (SIEST), Sungkyunkwan University, Suwon 440-746, Republic of Korea

[3]Department of Physics, University of Oxford, Clarendon Laboratory, Oxford, UK

[4]Pohang Accelerator Laboratory, Pohang, Kyungbuk 37673, Republic of Korea

[5]School of Chemical Engineering, Sungkyunkwan University, Suwon 440-746, Republic of Korea

Corresponding Authors: hshin@skku.edu; npark@skku.edu

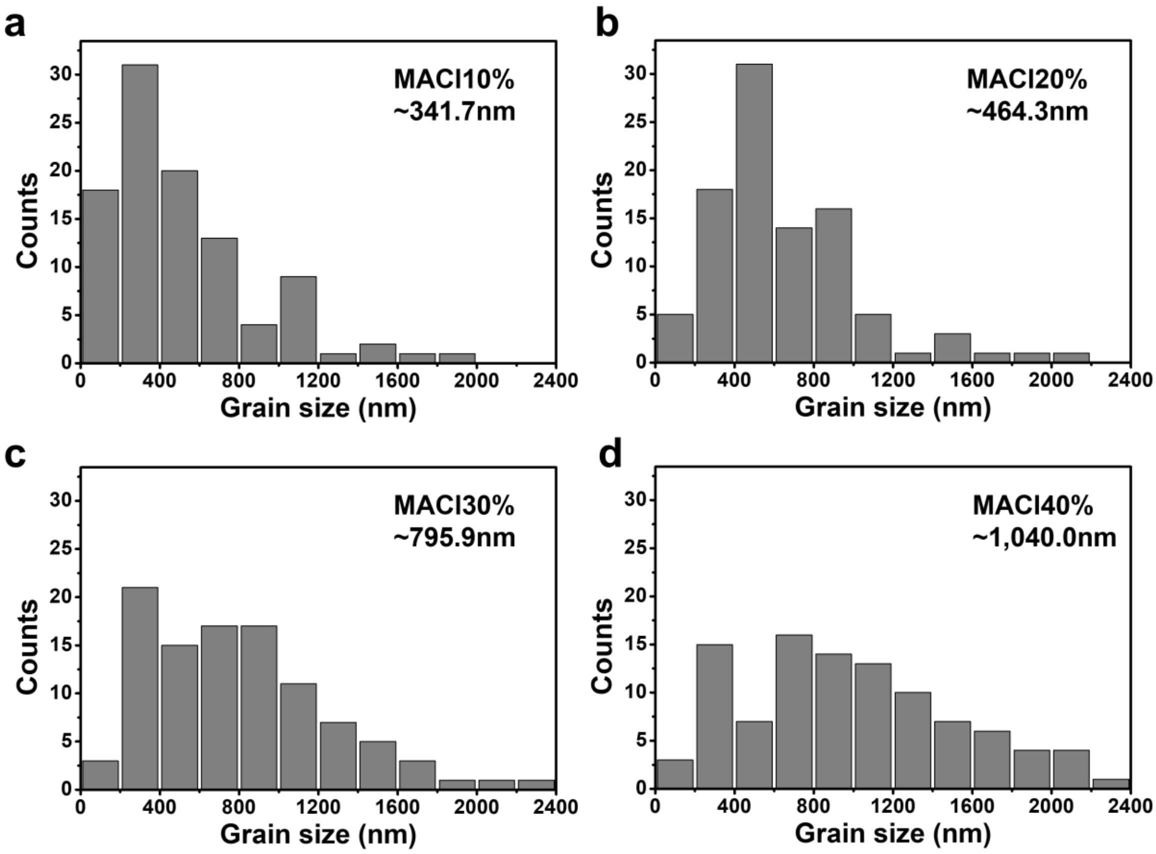

Fig. S 1 Grain size histograms showing the grain size distributions of approximately 100 grains obtained from SEM images of MACl10, 20, 30, and 40% thin films (**a, b, c,** and **d,** respectively). The average grain sizes of each thin film are ~341.7 nm (MACl10%), ~464.3 nm (MACl20%), ~795.9 nm (MACl30%), and ~1,040.0 nm (MACl40%).

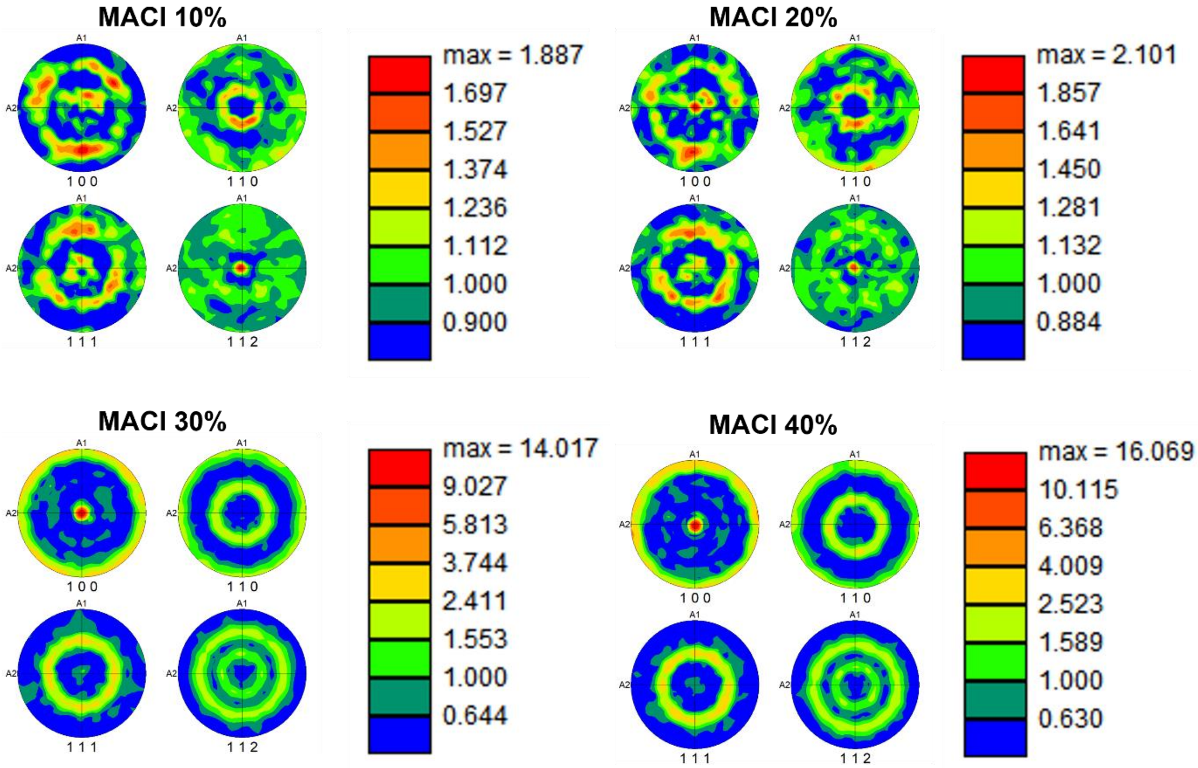

Fig. S 2 Inverse pole figures of MACl10, 20, 30, and 40% thin films. The (100), (110), (111), and (112) pole figures were derived from EBSD texture analyses from Fig. 1. When the max value is 1.000, the film is interpreted as being 'randomly' oriented.

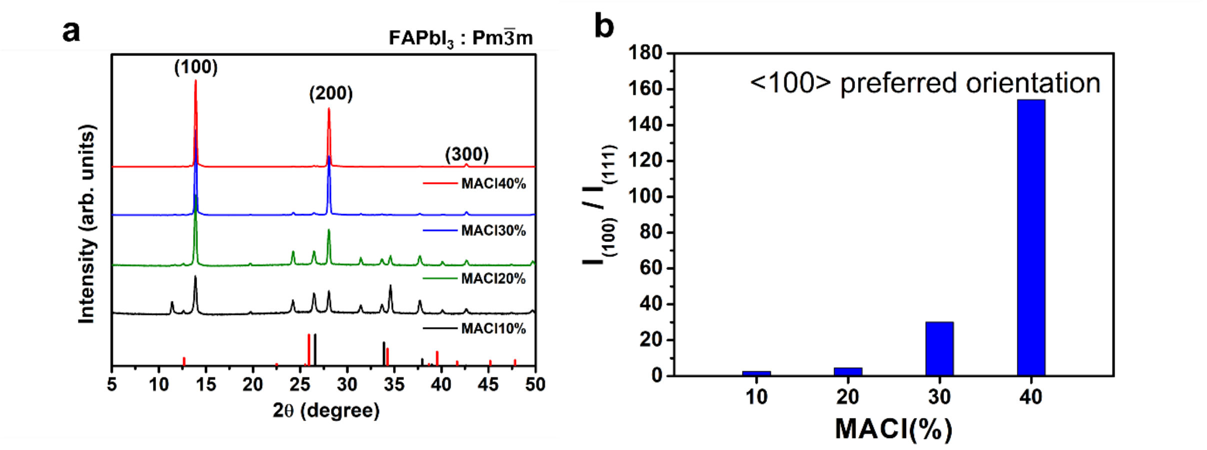

Fig. S 3 a, XRD patterns of MACl10, 20, 30, and 40% thin films. Red index peaks correspond to PbI_2, while black index peaks correspond to SnO_2 from the FTO substrate. XRD patterns show that all of the films form the α-$FAPbI_3$ phase, as the XRD patterns were fitted with the Pm$\bar{3}$m space group. **b,** (100) orientation degree calculated as the XRD peak intensity ratio of (100) and (111) from α-$FAPbI_3$.

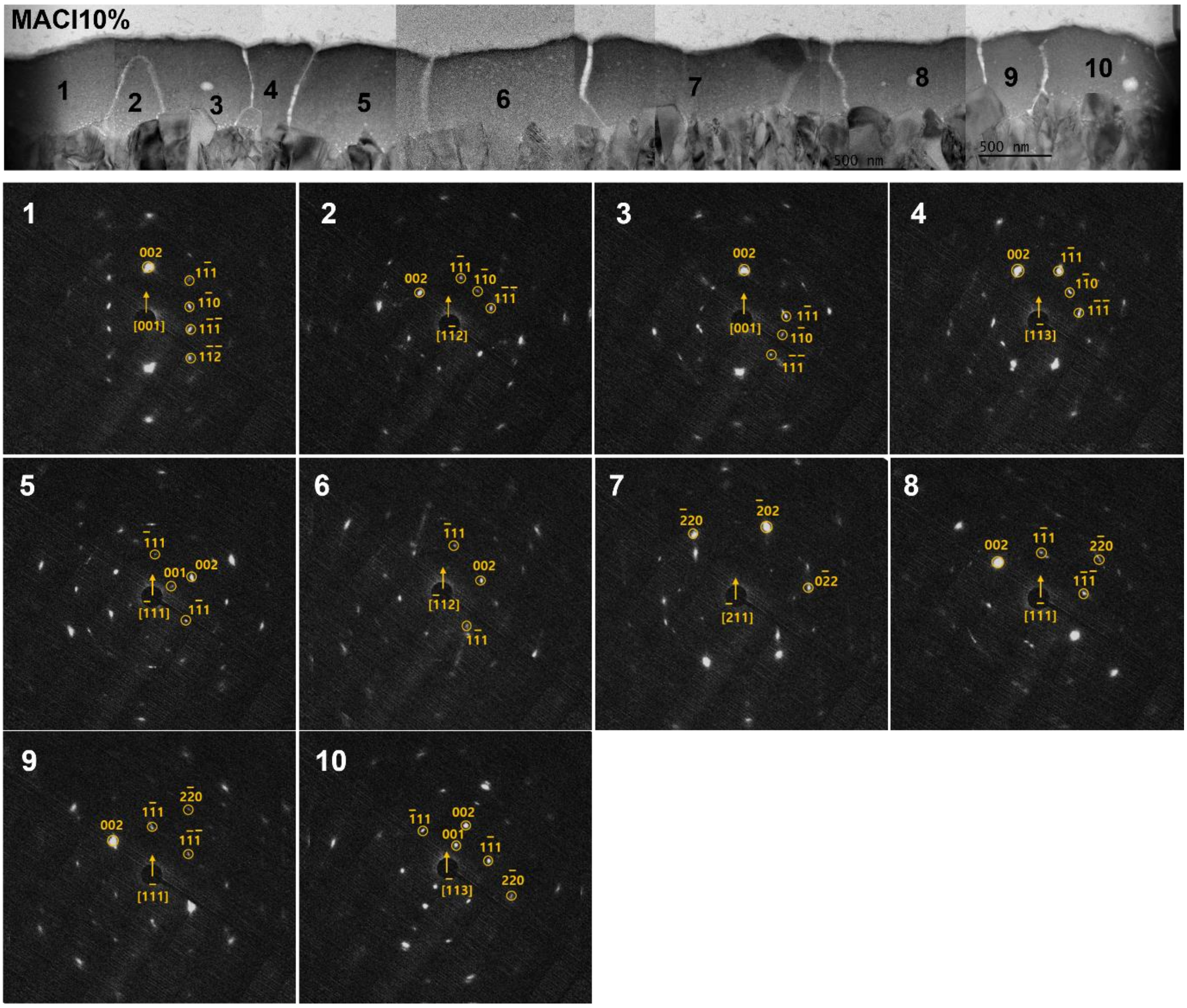

Fig. S 4 Cross-sectional HRTEM image of a MACl10% thin film and its corresponding SAED patterns taken from each grain (numbered 1~10). The HRTEM specimen was prepared using a FIB. The presence of the (100) plane along with the (200) plane shown in the SAED patterns indicates that MACl10% forms cubic α-$FAPbI_3$ in the $Pm\bar{3}m$ space group. The arrow marked in the SAED patterns indicates which axis is "face-up" relative to the substrate in each grain. Grains numbered 1 and 3 have the [100] axis "face-up" relative to the substrate, while the rest of the grains have the [111], [112], or [113] axis "face-up" relative to the substrate. This demonstrates that the growth alignment within the MACl10% thin film does not show a specific preferred orientation and is said to be a 'randomly oriented' growth.

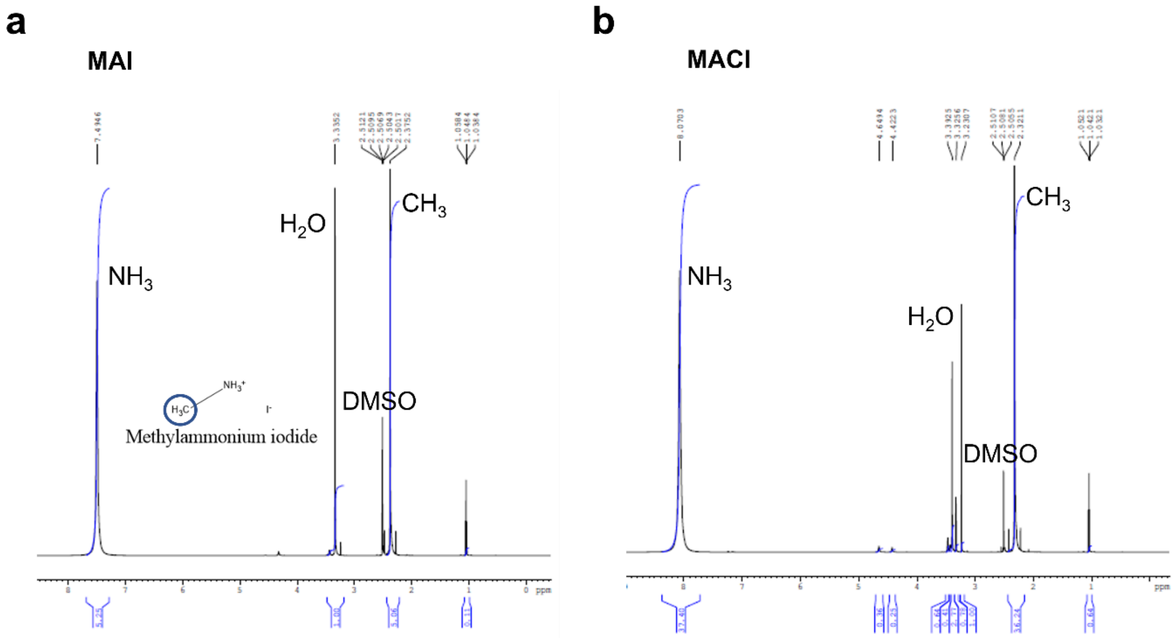

Fig. S 5 Liquid-state ^{1}H-NMR spectra of MAI and MACl. The peaks at 7.49 and 2.37 ppm correspond to N-*H* and C-*H* protons, respectively. The peaks at 2.5 and 3.33 ppm correspond to DMSO and water, respectively, from the NMR solvent.

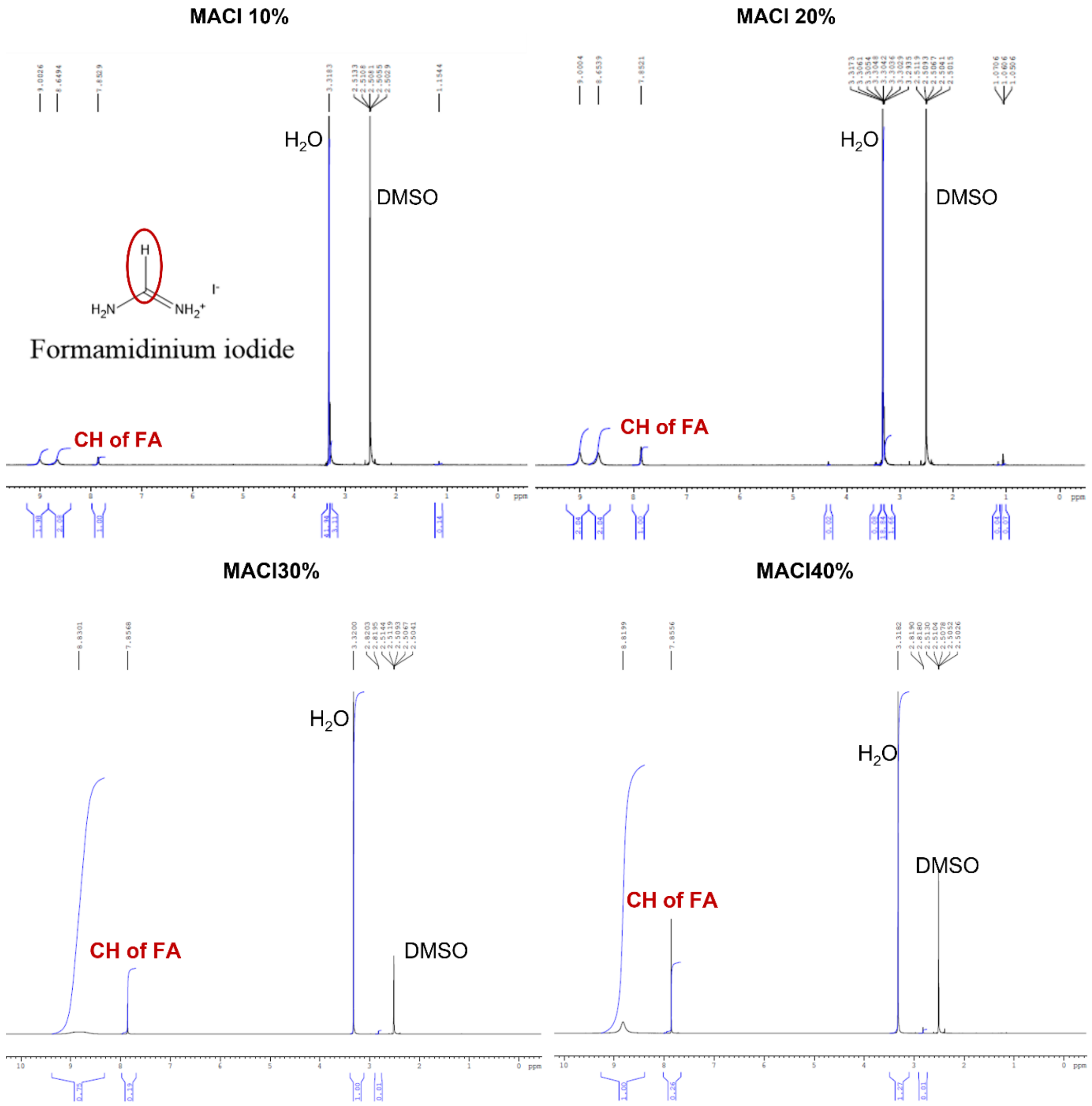

Fig. S 6 Liquid-state ^{1}H-NMR spectra of MACl10, 20, 30, and 40% thin films. As all of the thin films form α-FAPbI$_3$, the peak at 7.85 ppm corresponds to the C-*H* proton from FAI. The peaks at 2.5 and 3.32 ppm correspond to DMSO and water, respectively, from the NMR solvent. Compared to the ^{1}H-NMR spectrum of MACl shown in Fig. S5, neither the peak from the N-*H* proton nor that from the C-*H* proton in MACl was found throughout MACl10, 20, 30 and 40%. This indicates that even though the concentration of the MACl additive increased, the remaining MACl within α-FAPbI$_3$ was undetectable.

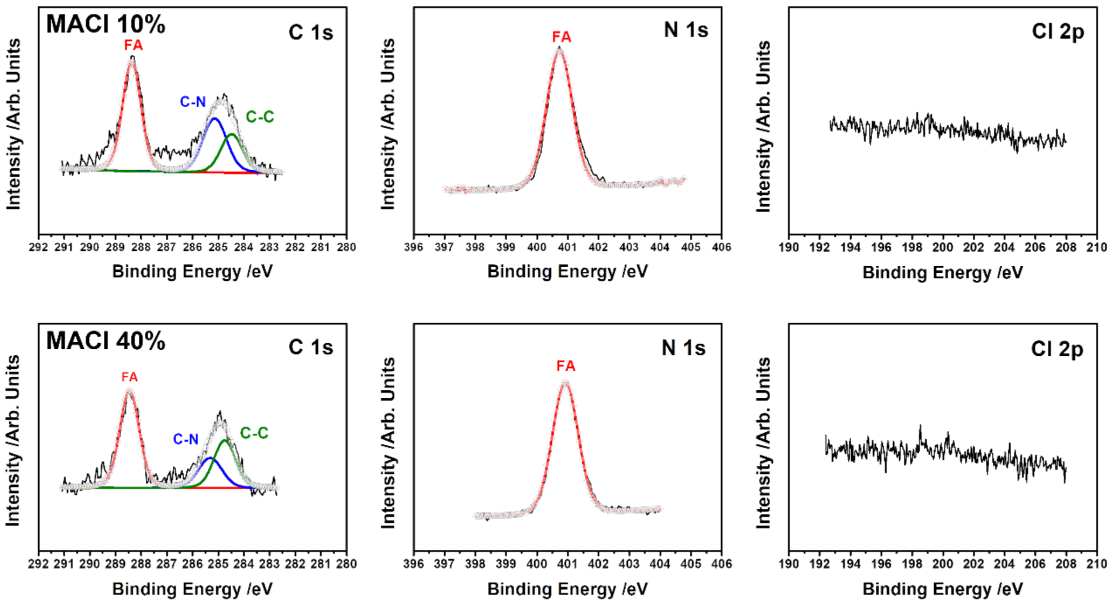

Fig. S 7 XPS spectra of MACl10 and 40% thin films. Both the MACl10% and MACl40% thin films show peaks corresponding to FA in the C 1s and N 1s peak regions. Although the concentration of MACl increases from 10% to 40%, there are no apparent peaks that correspond to MA in either the C 1s or N 1s peak regions. Additionally, no apparent peak was found in the Cl 2p peak region from either thin film.

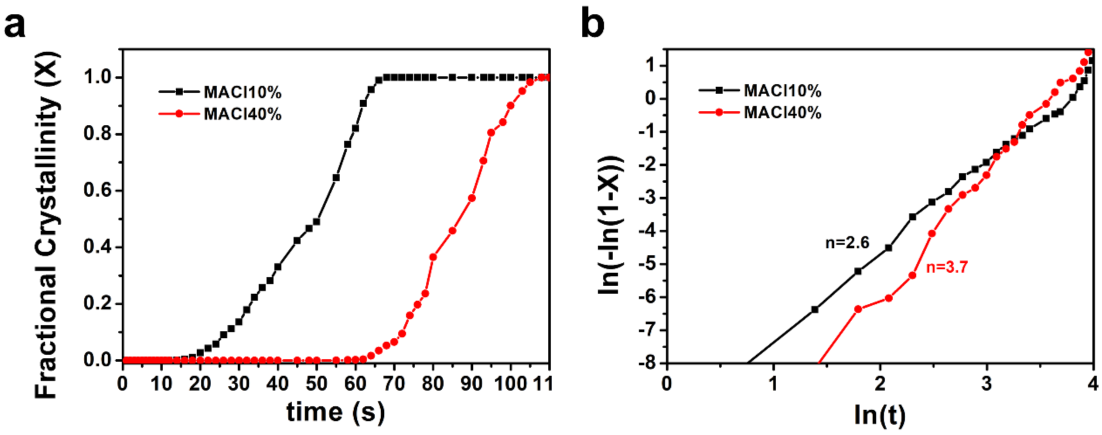

Fig. S 8 a, Fractional crystallization curves for the isothermal crystallization process of $FAPbI_3$ solutions with MACl10% and MACl40% at 100°C. The solutions were dropped onto substrates and then spin coated at 6000 rpm for 15 sec. The crystallization process was recorded after the substrates were transferred to a hot plate. We note that to observe the effect of the MACl concentration on the crystallization process, an anti-solvent was not applied. The anti-solvent method induces fast nucleation. Nucleation and growth were detected after 12 and 55 sec for MACl10% and MACl40%, respectively. The fractional crystallization reached 1.0 after 66 and 108 sec for MACl10% and MACl40%. **b,** Avrami plot of the fractional crystallization (X) over time. The Avrami exponent was obtained from the linear slope, n.

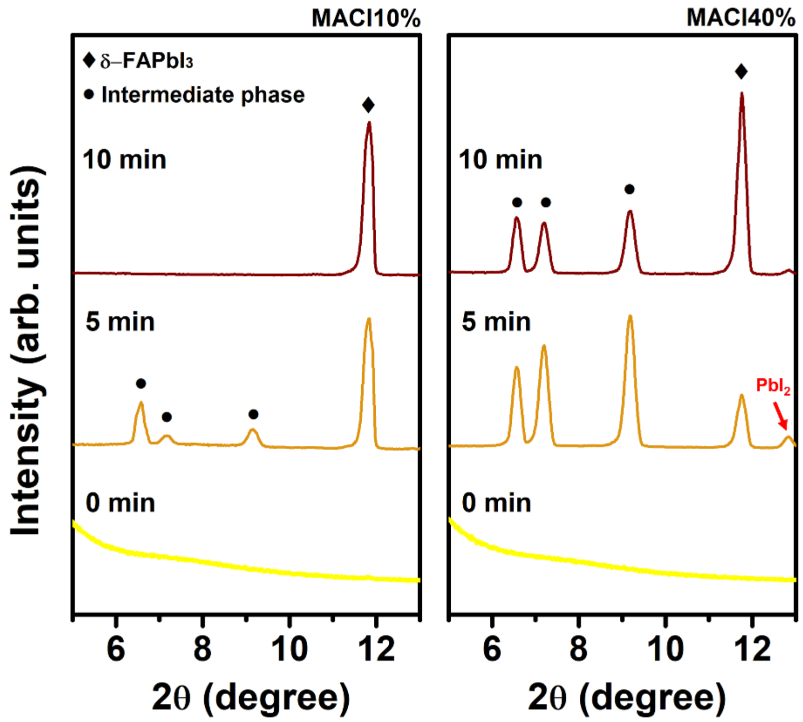

Fig. S 9 *Ex-situ* XRD patterns of both MACl10% and MACl40% measured throughout the crystallization process at room temperature. With the duration time of 5 min, each XRD patterns were measured right after spin coating the precursor solution onto the FTO substrate. Due to no heat treatment to induce slower crystallization, both MACl10 and 40% has resulted in δ-phase $FAPbI_3$. Along with the peaks of the intermediate phase, (001) peak of 2H PbI_2 phase (red) also appears during the process. MACl40% results in slower reaction towards the intermediate phase to the crystalline $FAPbI_3$.

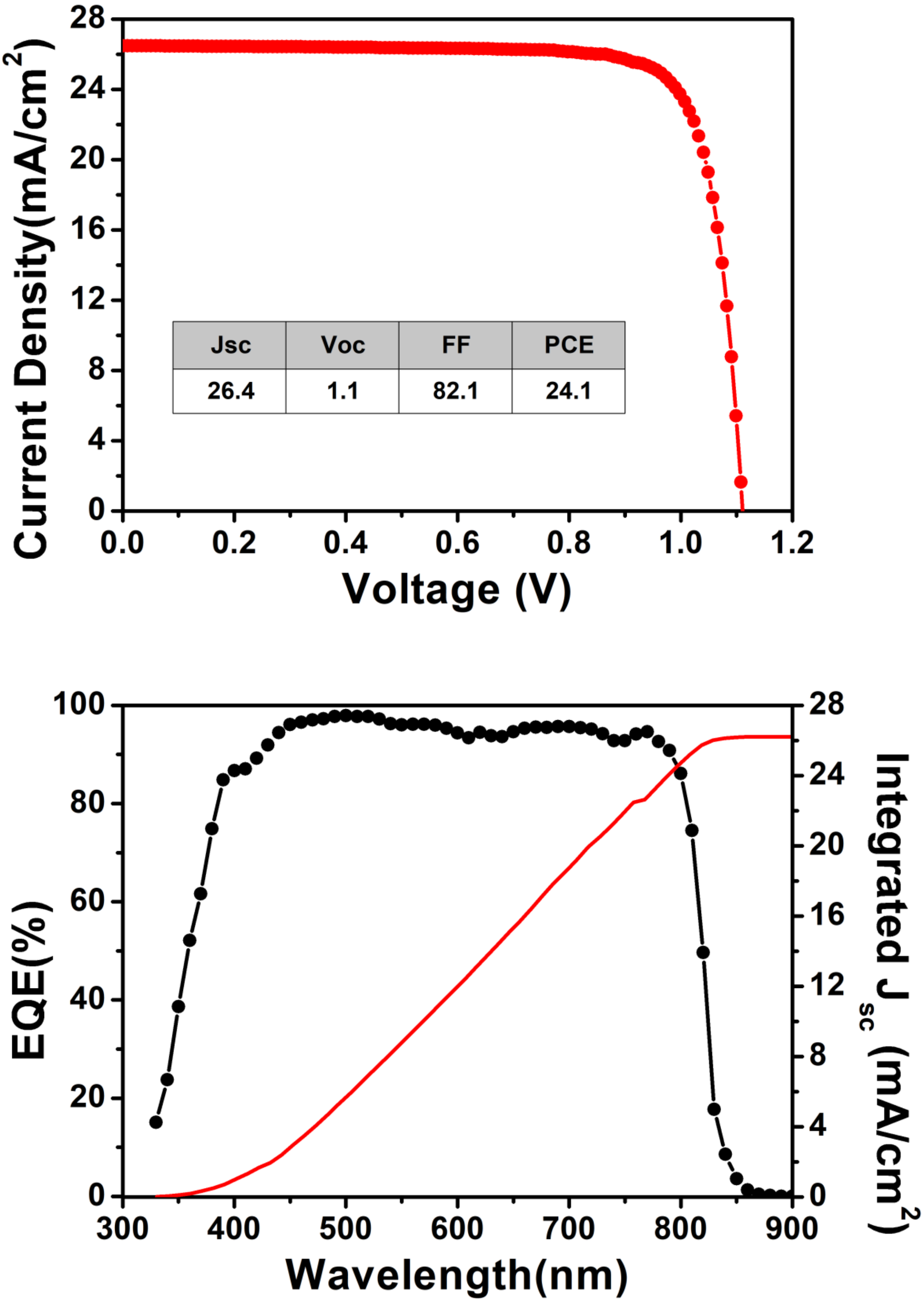

Fig. S 10 Reverse scan J-V curve and EQE spectra of the champion device with 4MEO-PEAI passivation layer.

The fabricated structure contains anti-reflective film/ FTO/ALD-SnO_2/SnO_2 NP/α-$FAPbI_3$/4MEO-PEAI/Spiro-OMeTAD/Au. Photovoltaic parameters of of J_{sc}, V_{oc}, FF and PCE of the champion device are inserted in the plot. The integrated J_{sc} was extracted from the EQE spectrum. Bandgap energy taken from the EQE spectrum at the inflection point of the absorption threshold is 1.51 eV.

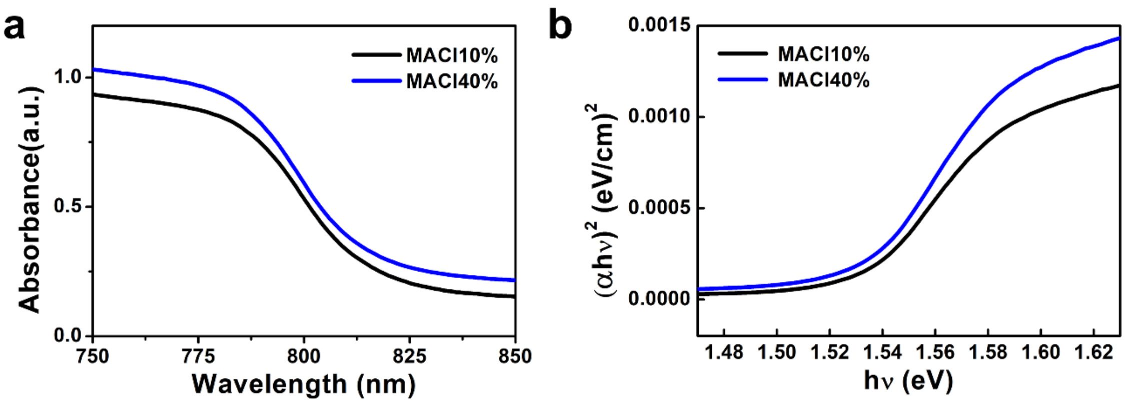

Fig. S 11 a, UV–vis absorption spectra of MACl10 (black) and 40% (blue) thin films. **b,** Tauc plot of $(\alpha h\nu)^2$ vs. hν for the absorption spectra. Although the absorption edge and the optical bandgap of MACl10% and MACl40% seem similar, the MACl40% thin film shows a slight redshift.

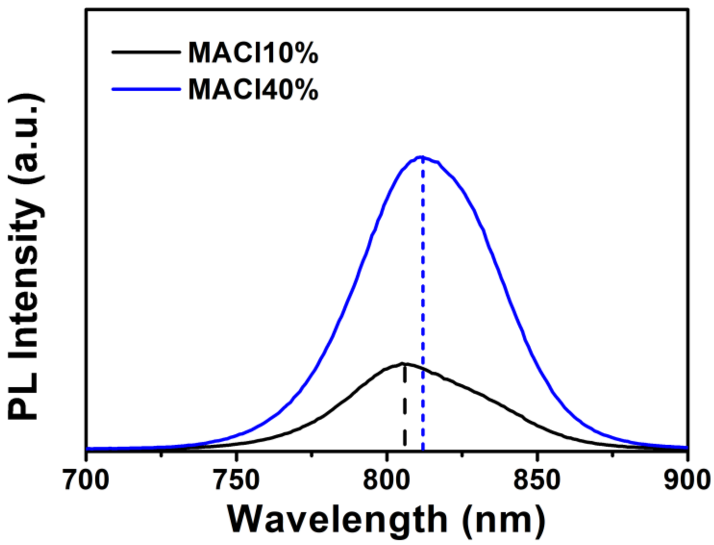

Fig. S 12 PL spectra of MACl10 (black) and 40% (blue) thin films. The PL peak position shifts from 806 nm (MACl10%) to 812 nm (MACl40%) with an increase in the absorbance.

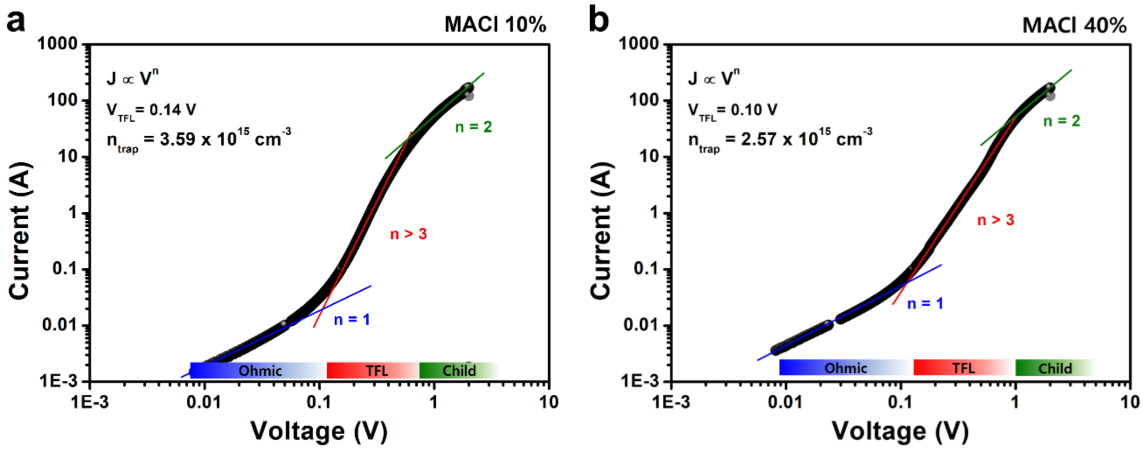

Fig. S 13 SCLC measurements of MACl10 and 40%. An electron-only device was fabricated with the structure FTO/ALD-SnO_2/SnO_2 NPs/perovskite/PCBM/Au. The current-voltage responses show three different dependence regions: a linear ohmic region (n=1, blue), a trap-filling region (n>3, red) and a Child's region (n=2, green). The electron trap-state density (n_{trap}) of both samples was calculated.

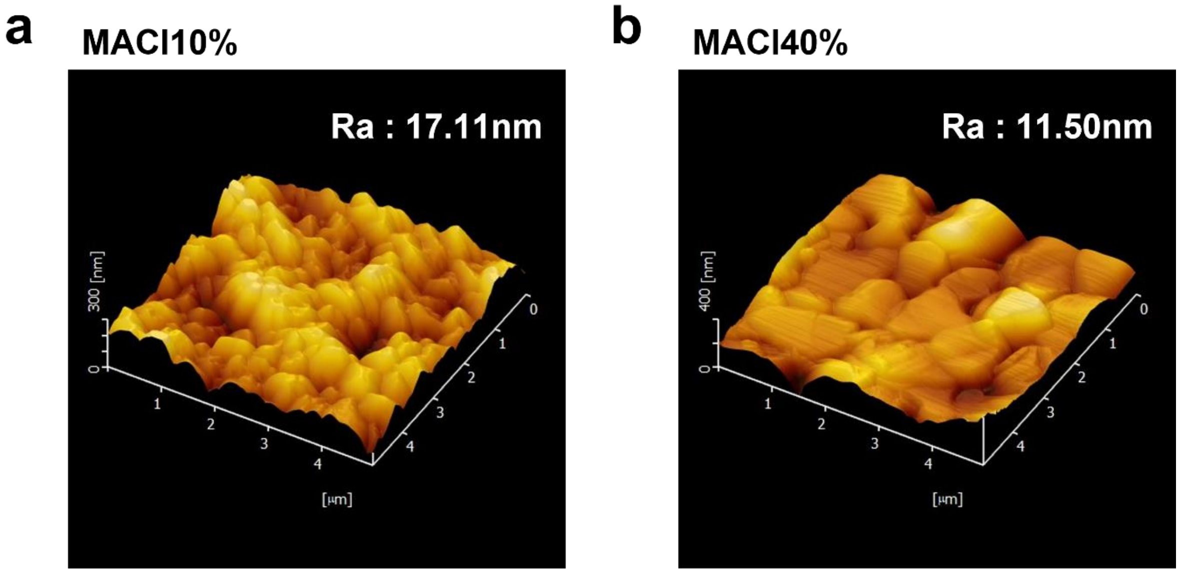

Fig. S 14 Surface roughness measured by AFM. 3D topography image of **a,** a MACl10% thin film and **b,** a MACl40% thin film in the same range (5 µm x 5 µm). The average roughness (Ra) of the entire measured area is higher in MACl10% (17.11 nm) than in MACl40% (11.50 nm). This is due to not only the grain size difference between the two thin films but also the flatness of the grain surfaces.

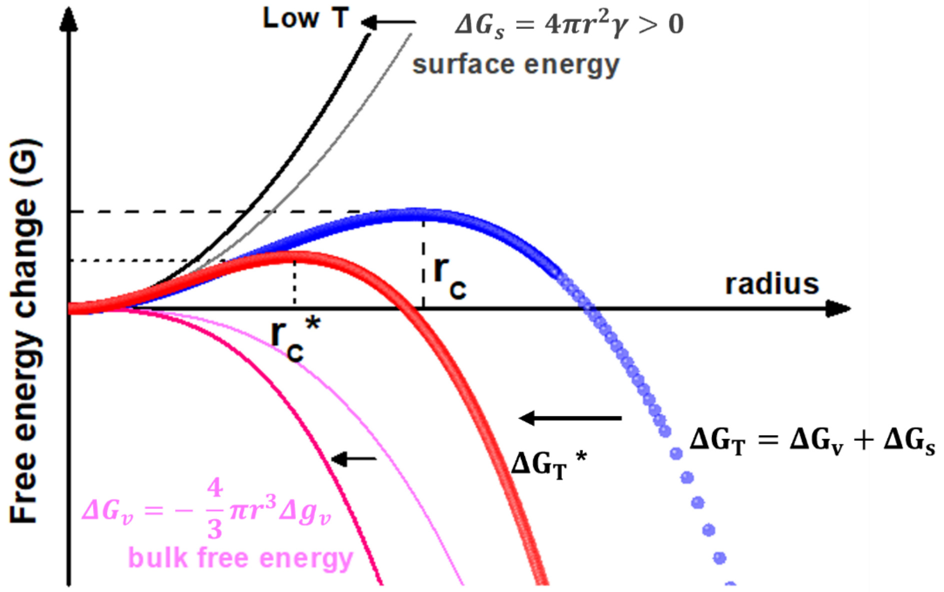

Fig. S 15 Gibbs free energy (G_T) as a function of the nucleus radius. G_T consists of a volumetric term G_V and a surface term G_S, where G_v is the Gibbs free energy per volume and $\boldsymbol{\gamma}$ is the surface tension. The critical radius can be determined from G_T, as it is the minimum nucleus size to start the nucleation process. With lower temperature, G_T decreases as G_V, which depends on temperature, decreases.

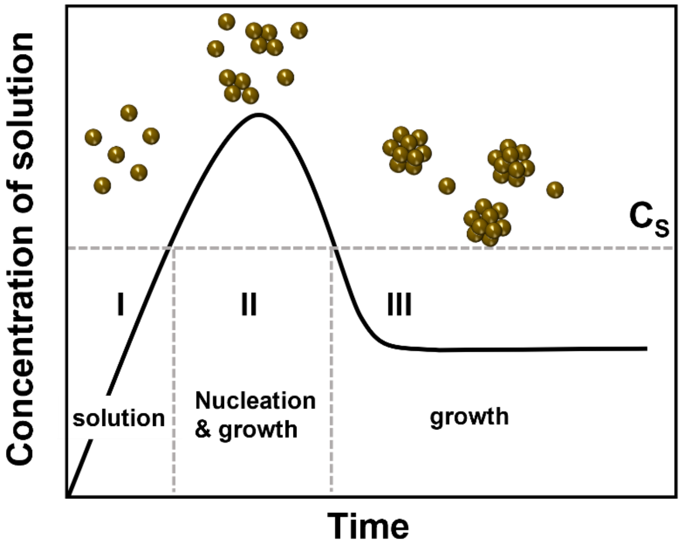

Fig. S 16 Conventional LaMer's model: concentration change in the α-$FAPbI_3$ precursor solution as a function of time. C_S is the supersaturation concentration of the precursor solution. Formation of α-$FAPbI_3$ nuclei starts to occur in stage I, and nucleation continues to occur in stage II. As the consumption of the solute becomes faster than the evaporation of the solvent, the solution concentration decreases to below C_S, resulting in growth of the nuclei in stage III.

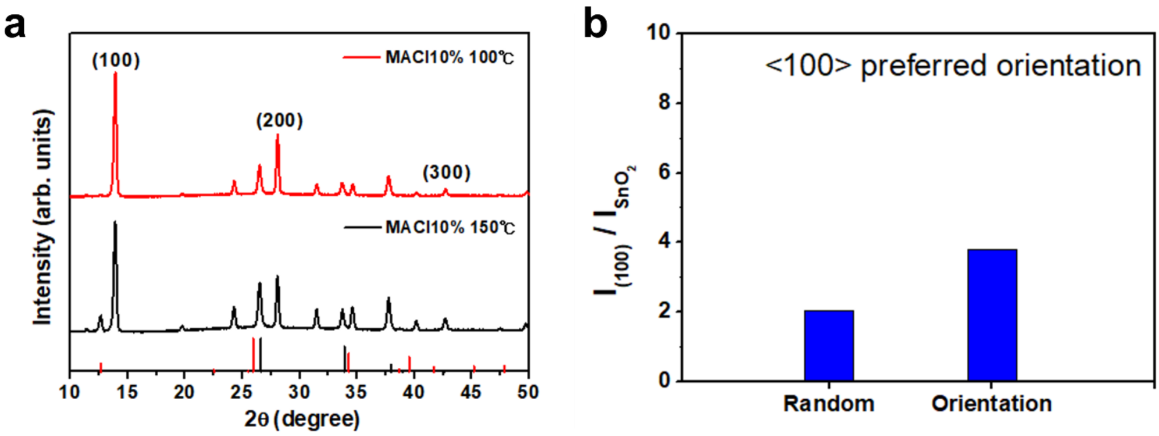

Fig. S 17 a, XRD patterns of MACl10% (150℃) and MACl10% (100℃) thin films. Red index peaks correspond to PbI_2, while black index peaks correspond to SnO_2 from the FTO substrate. The XRD patterns show that both thin films form the α-$FAPbI_3$ phase. **b,** (100) orientation degree calculated as the XRD peak intensity ratio of (100) from α-$FAPbI_3$ and (110) from SnO_2 as a reference.

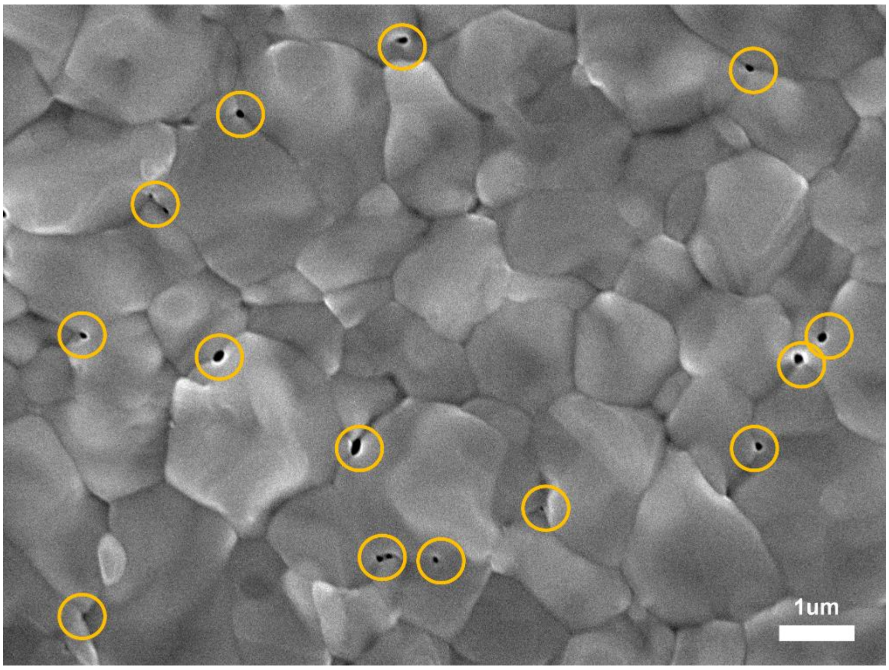

Fig. S 18 SEM image of MACl40% thin film fabricated by 2-step heat treatment. The average grain size of ~2,2 μm was obtained. The pin holes were found between the grains (marked by the yellow circles).

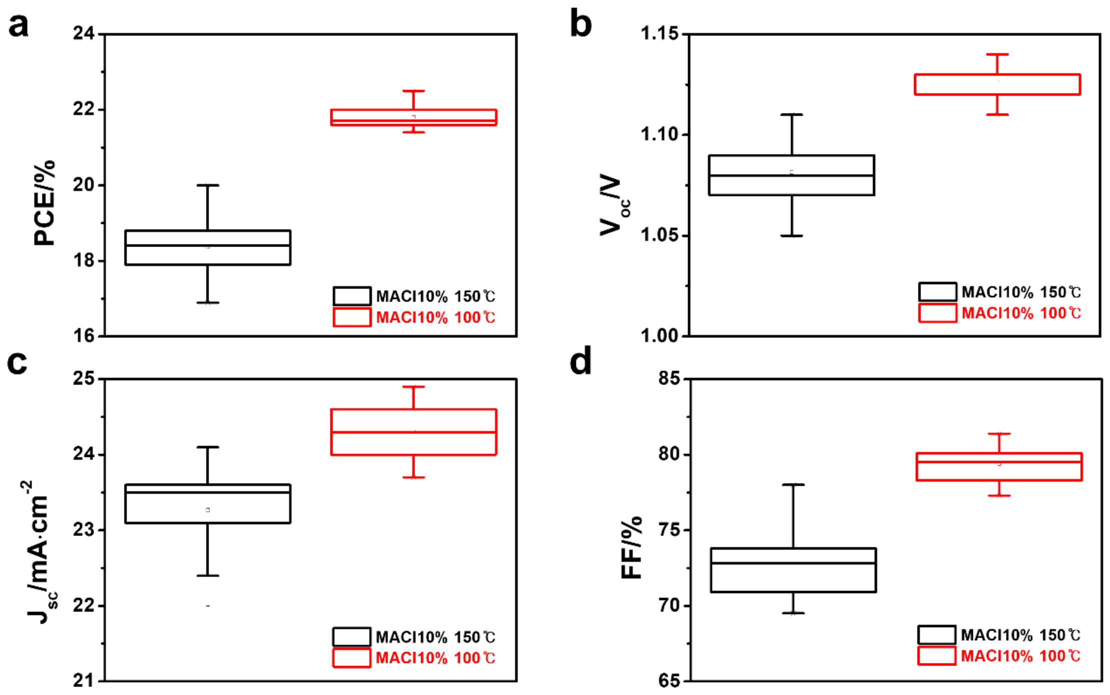

Fig. S 19 Box charts of statistical PV parameters for α-$FAPbI_3$-based PSCs of MACl10% (150℃) and MACl10% (100℃). (The parameters of MACl10% (150℃) were taken from Figs. 2a-d for comparison.)

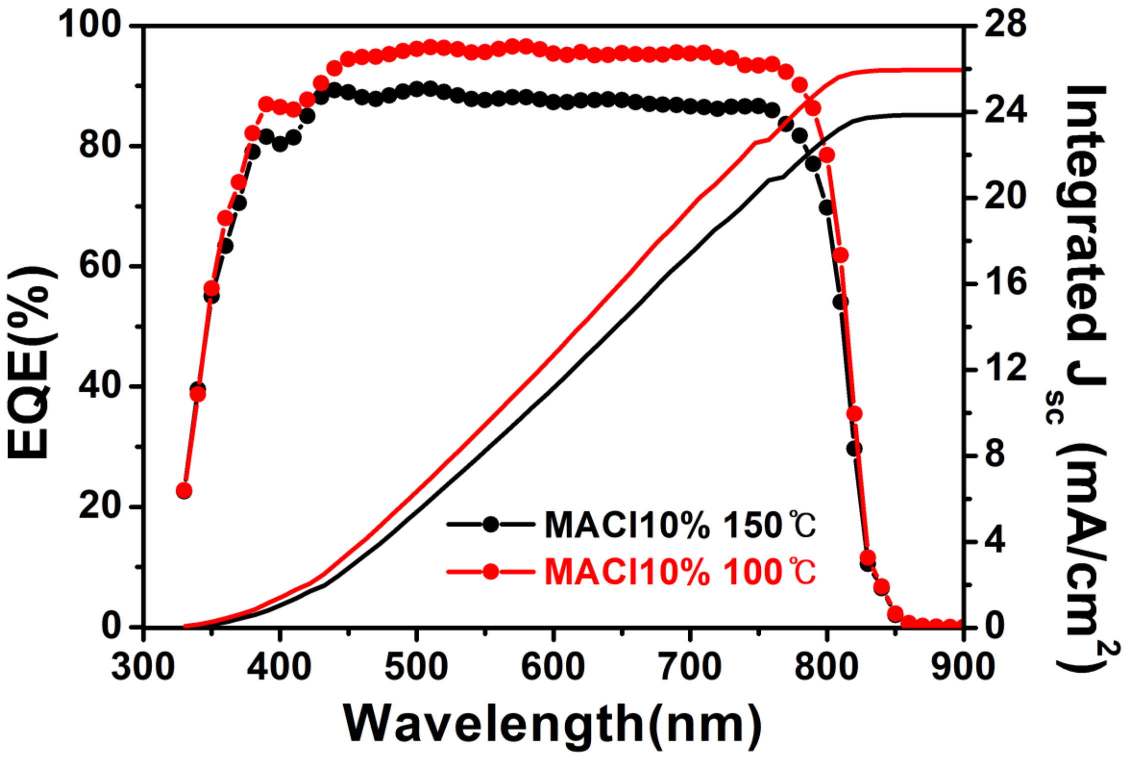

Fig. S 20 EQE spectra of α-$FAPbI_3$-based PSCs of MACl10% (150℃) and MACl10% (100℃). (The EQE spectrum of MACl10% (150℃) was taken from Fig. 2e for comparison.) The integrated J_{sc} was extracted from the EQE spectrum.
